# The Sphingosine and Phytosphingosine Ceramide Ratio in Lipid Models Forming the Short Periodicity Phase: An Experimental and Molecular Simulation Study

**DOI:** 10.1021/acs.langmuir.4c00554

**Published:** 2024-06-25

**Authors:** Andreea Nădăban, Chloe O. Frame, Dounia El Yachioui, Gerrit S. Gooris, Robert M. Dalgliesh, Marc Malfois, Christopher R. Iacovella, Annette L. Bunge, Clare McCabe, Joke A. Bouwstra

**Affiliations:** † Division of BioTherapeutics, Leiden Academic Centre for Drug Research, Leiden University, Leiden 2333CC, The Netherlands; ‡ Department of Chemical and Biomolecular Engineering, Vanderbilt University, Nashville, Tennessee 37235-1604, United States of America; § ISIS Neutron and Muon Source, Science and Technology Facilities Council, Rutherford Appleton Laboratory, Didcot OX11 0QX, United Kingdom; ∥ ALBA Synchrotron, Cerdanyola del Vallès, 08290 Barcelona, Spain; ⊥ Department of Chemical and Biological Engineering, Colorado School of Mines, Golden, Colorado 80401, United States of America; # School of Engineering and Physical Science, Heriot-Watt University, Edinburgh EH14 4AS, United Kingdom

## Abstract

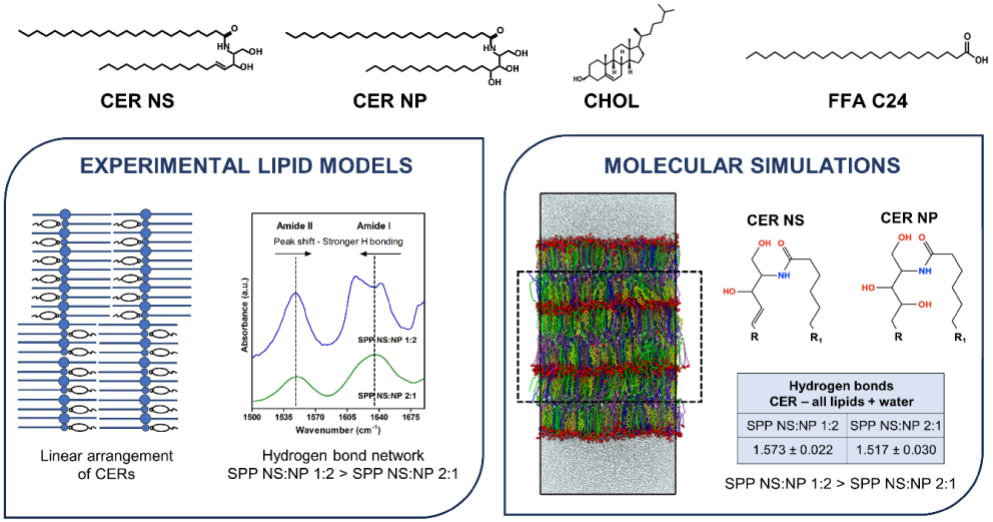

The lipids located in the outermost layer of the skin, the stratum corneum (SC), play a crucial role in maintaining the skin barrier function. The primary components of the SC lipid matrix are ceramides (CERs), cholesterol (CHOL), and free fatty acids (FFAs). They form two crystalline lamellar phases: the long periodicity phase (LPP) and the short periodicity phase (SPP). In inflammatory skin conditions like atopic dermatitis and psoriasis, there are changes in the SC CER composition, such as an increased concentration of a sphingosine-based CER (CER NS) and a reduced concentration of a phytosphingosine-based CER (CER NP). In the present study, a lipid model was created exclusively forming the SPP, to examine whether alterations in the CER NS:CER NP molar ratio would affect the lipid organization. Experimental data were combined with molecular dynamics simulations of lipid models containing CER NS:CER NP at ratios of 1:2 (mimicking a healthy SC ratio) and 2:1 (observed in inflammatory skin diseases), mixed with CHOL and lignoceric acid as the FFA. The experimental findings show that the acyl chains of CER NS and CER NP and the FFA are in close proximity within the SPP unit cell, indicating that CER NS and CER NP adopt a linear conformation, similarly as observed for the LPP. Both the experiments and simulations indicate that the lamellar organization is the same for the two CER NS:CER NP ratios while the SPP NS:NP 1:2 model had a slightly denser hydrogen bonding network than the SPP NS:NP 2:1 model. The simulations show that this might be attributed to intermolecular hydrogen bonding with the additional hydroxide group on the headgroup of CER NP compared with CER NS.

## Introduction

Ceramides (CERs) represent one of the main lipid classes of the intercellular regions of the outermost layer of the skin, the stratum corneum (SC). CERs belong to the group of sphingolipids, an important component of biological membranes, which are involved in different biological processes like cell proliferation, differentiation, and apoptosis.^1,2^ CERs are composed of a sphingolipid base linked by an amide bond to an acyl chain, with 24 CER subclasses identified in human SC.^3^ An important and characteristic subclass of CERs in the SC are CERs with a very long ω-hydroxy acyl chain linked with an ester bond to an acyl chain.^4^ In human SC, CER NP (with a phytosphingosine chain lined to a nonhydroxy acyl chain) is one of the most abundant CER subclasses.^5−8^ Another important CER subclass is CER NS, with a sphingosine linked to a nonhydroxy acyl chain. CERs, along with cholesterol (CHOL) and free fatty acids (FFAs), form the highly organized lipid matrix of the SC, which restricts water loss and prevents the permeation of pathogens and other hazardous materials into the body.^4,9−11^ The SC lipid matrix does not include phospholipids, in contrast with other biological membranes.^12^

The organization of SC lipids is different from other membranes with high CHOL content, in which a liquid ordered phase is often present.^13^ Lipids in human SC are organized into two coexisting crystalline lamellar phases with repeat distances of ∼13 nm and ∼6 nm called the long and short periodicity phases (LPP and SPP), respectively.^14−16^ Previous studies have shown that the esterified ω-hydroxy sphingosine ceramide (CER EOS) plays an important role in the formation of the LPP.^17,18^ When CER EOS is not present in the lipid model systems, only the SPP is formed. A gradual increase in CER EOS concentration enhances the formation of the LPP in SC lipid model systems and in SC at the expense of the SPP.^3,19,20^

In the LPP and SPP, the lipid chains can have different packing densities within the lamellae, referred to as the lateral organization: orthorhombic (ordered phase, very dense lipid packing), hexagonal (ordered phase, but less dense packing), or liquid phase (disordered lipid packing).^21−23^ The human SC lipids mainly adopt an orthorhombic packing, with a smaller fraction of lipids forming a hexagonal phase. We note that small liquid domains have been reported in human SC.^24^

The SC lipids play a crucial role in the skin barrier function.^10,25^ Changes in the SC lipid composition have been reported in inflammatory skin diseases like psoriasis, atopic dermatitis, or seborrheic dermatitis.^25−32^ Among the different lipid compositional changes, an elevated molar ratio of CER NS:CER NP is often encountered in diseased SC and reported to correlate with an impaired skin barrier.^30,32,33^ The effect of the CER NS:CER NP molar ratio on the lipid organization and barrier function was recently studied using a lipid model that formed exclusively the LPP.^34^ The aim of the present study was to investigate the effect of a variation of this ratio in a lipid model in which the lipids form only the SPP (in the absence of CER EOS) using experiments and molecular dynamics (MD) simulations.

Previously, it was shown that lipid models consisting of isolated CERs (extracted from porcine or human SC) mixed with CHOL and FFAs can mimic the unique lamellar organization of the SC lipids.^35−37^ Moreover, replacing the isolated porcine or human CERs with their synthetic counterparts did not affect the lamellar phase behavior of the lipid models, which still mimicked the native SC lipid organization.^19,20,38−40^ The CER composition of lipid models forming only the SPP varied in different studies from a mix of CER subclasses alongside CHOL and FFAs, to systems that only included a single CER subclass.^41−51^ A similar lamellar and lateral organization of the lipids was reported for the models prepared with a CER subclass composition mimicking that in porcine SC and models with only CER NS, mixed with CHOL and FFAs.^41−46^ Using lipid models with a limited number of components offers the opportunity of a more detailed analysis due to the availability of deuterated lipids and also the opportunity to perform MD simulations, which can provide molecular level insights into the experimental observations. In particular, MD simulations are useful for studying the molecular level structure of the SC lipids.^52−55^

To date, most MD simulations of SC lipids have used fully atomistic models, in which all the atoms in the system are explicitly represented, and consider only bilayer systems in water because of the computational cost of studying larger multilayer systems.^52^ Coarse-grained (CG) models, in which a group of atoms is described by a single interaction site (a CG bead), can be used to study multilamellar systems and have been developed for several SC lipids.^52^ The simplified representation of atoms used in CG models substantially reduces the computational cost by allowing larger time steps of integration and speeding up the dynamics of the system.^56,57^ As a result, CG models can access longer timescales and larger system sizes than atomistic models enabling simulations to examine self-assembly of the multilayer structures required for CER molecules to adopt linear conformations (in which the acyl chain and sphingosine base are positioned on each side of the headgroup) that occur in SC lipid model systems.^45,58^ However, the atomic-level detail provided by atomistic simulations is necessary for calculating properties such as hydrogen bonding. Thus, a multiscale approach that combines the efficiency of CG models with the details of atomistic models is necessary for a robust study of SC lipid model behavior. This combined multiscale strategy has been used in prior studies of SC lipids to self-assemble multilayer SC lipid systems, which were then reverse-mapped to recover the atomistic details required to study hydrogen bonding, density profiles, and neutron scattering behavior in comparison with experiments.^54,58,59^

In this study, we used a system of CER NS, CER NP, CHOL, and lignoceric acid (FFA C24) with CER NS:CER NP molar ratios of 1:2 (mimicking the ratio observed in healthy SC) and 2:1 (an approximate ratio corresponding to inflammatory skin diseases). Experimentally, the lamellar organization, lipid packing, lipid chain interactions, and hydrogen-bond network in the systems were examined. To provide more details, MD simulations were performed on lipid compositions comparable to those in the experiments. CG models were self-assembled into multilayer systems, reverse-mapped back to the atomistic level, and analyzed to determine structural metrics (bilayer height, area per lipid, chain tilt angle, and nematic order parameter), neutron scattering length density profiles, and hydrogen bonding; results were compared with the experiments. Combining simulation and experimental data provides a more comprehensive view of the molecular-level arrangement of the SPP.

## Materials and Methods

### Experimental Materials and Methods

#### Materials

The CERs used in this study were *N*-(tetracosanoyl)-sphingosine (CER NS C24) and *N*-(tetracosanoyl)-phytosphingosine (CER NP C24), kindly donated by Evonik (Essen, Germany). These two CERs with perdeuterated acyl chains (CER NSd47 and CER NPd47) (Figure S1.1) were also used. CER NS with the sphingosine chain terminally deuterated (CER NSd7) was acquired from Avanti Polar Lipids (Alabama, USA). The sphingoid bases of CER NS and CER NP had a chain length of 18 carbon atoms. CHOL, FFA C24, D_2_O, and the acetate buffer salts were obtained from Sigma-Aldrich-Chemie GmbH (Schnelldorf, Germany). The perdeuterated FFA C24 (DFFA C24) was purchased from Arc Laboratories B.V. (Apeldoorn, The Netherlands). All organic solvents were of analytical grade, acquired from Biosolve B.V. (Valkenswaard, The Netherlands). The Nuclepore track-etched membranes were purchased from Whatman (Kent, UK). The Milli-Q water was of Millipore quality.

#### Lipid Model Compositions and Preparation

The lipid models were prepared with an equimolar ratio of CERs, CHOL, and FFA C24. Two molar ratios of CER NS:CER NP were included in this study: 1:2 and 2:1 (Table 1). For the Fourier-transform infrared spectroscopy (FTIR) studies, similar models were prepared with DFFA C24, CER NSd47, and/or CER NPd47. For neutron diffraction studies, a model with CER NSd7 was prepared (SPP NS:NP 2:1). All of the models studied are presented in Table 1.

**Table 1:** Sample Compositions and Abbreviations of the Lipid Models Studied Experimentally

Lipid model	Composition	Molar ratios
SPP NS:NP 1:2	CER NS C24: CER NP C24: CHOL: FFA C24	0.33:0.66:1:1
SPP NS:NP 2:1	CER NS C24: CER NP C24: CHOL: FFA C24	0.66:0.33:1:1
SPP NSd47:NPd47:DFFA 1:2	CER NSd47: CER NPd47: CHOL: DFFA C24	0.33:0.66:1:1
SPP NSd47:NPd47:DFFA 2:1	CER NSd47: CER NPd47: CHOL: DFFA C24	0.66:0.33:1:1
SPP NS:NPd47:DFFA 1:2	CER NS: CER NPd47: CHOL: DFFA C24	0.33:0.66:1:1
SPP NS:NPd47:DFFA 2:1	CER NS: CER NPd47: CHOL: DFFA C24	0.66:0.33:1:1
SPP NSd47:NP:DFFA 1:2	CER NSd47: CER NP: CHOL: DFFA C24	0.33:0.66:1:1
SPP NSd47:NP:DFFA 2:1	CER NSd47: CER NP: CHOL: DFFA C24	0.66:0.33:1:1
SPP NSd7:NP 2:1	CER NSd7: CER NP: CHOL: FFA C24	0.66:0.33:1:1

The required amount of each individual lipid was dissolved in chloroform:methanol (2:1, v/v) at a concentration of 5 mg/mL. For the samples used for FTIR studies, 1 mg of the lipid composition was sprayed onto a AgBr window over a 10 mm × 10 mm area. The samples for X-ray studies were dissolved in hexane:ethanol (2:1, v/v) and then sprayed onto a Nuclepore polycarbonate membrane, over an area of 2 mm × 3 mm. During spraying, a Camag Linomat IV sprayer (Muttenz, Switzerland) was used with a spraying rate of 14 s/μL. For the neutron diffraction measurements, 10 mg of lipids dissolved in chloroform:methanol (2:1, v/v) were sprayed onto a silicon substrate over an area of 1.2 cm × 3.8 cm using the same spraying conditions. All samples were equilibrated at 95 °C for 65 min, then slowly cooled to room temperature for over 50 min. Lastly, the samples were hydrated with either deuterated acetate buffer (pH 5.0, for the FTIR studies) or D_2_O/H_2_O buffer (at three ratios, 8%, 50%, and 100% D_2_O) for the neutron studies. This hydration occurred at 37 °C for at least 12 h. Prior to the X-ray measurements, the lipid samples were maintained at 80% relative humidity for at least 24 h.

#### FTIR Measurements

The FTIR data were collected on a PerkinElmer Frontier FTIR (PerkinElmer, Waltham, USA), with a nitrogen-cooled mercury cadmium telluride detector. The sample compartment was purged with a continuous flow of dry air to remove moisture. Each spectrum consists of 77 interferograms with a resolution of 1 cm^–1^. The samples were measured in the wavenumber range of 500–4000 cm^–1^, between 10 and 90 °C at a heating rate of 4 min/°C. The spectra were extracted using TimeBase (PerkinElmer, Waltham, USA) and processed using Spectrum (PerkinElmer, Waltham, USA). The data were deconvoluted using γ = 2.2 and a smoothing factor of 76.7. Three measurements were performed for each composition included in this study.

By analyzing the CH_2_ symmetric stretching vibrations (ν_s_CH_2_), information about the lateral packing and the phase transition of the lipid chains was acquired. The ν_s_CH_2_ vibrations are observed at ∼2849 cm^–1^ and the CD_2_ symmetric stretching vibrations at ∼2090 cm^–1^ (ν_s_CD_2_). The mid-phase transition temperature is defined as the temperature where the lipids are transitioning from orthorhombic to hexagonal packing (*T*_m_O-H) or from hexagonal to liquid (*T*_m_H-L) packing. It was calculated by using the linear regression curve fitting method described elsewhere.^44^ The lipid chain packing is determined by examining the CH_2_ and CD_2_ scissoring vibrations (δCH_2_, wavenumber range: 1462–1473 cm^–1^; δCD_2_, wavenumber range: 1085–1095 cm^–1^). Python scripts were used for the determination of the δCH_2_ and δCD_2_ peak positions (fitting Lorentzian peaks) and peak heights. A peak height ratio (OR/MID) was then calculated as the ratio of the average peak height of the two orthorhombic peaks and the height of the central peak. Statistical analyses were performed using GraphPad Prism (v.8) to determine the statistical significance of the mid-phase transition temperatures and scissoring peak splitting of the different compositions (unpaired *t* test, significance level set at *p* < 0.05). For the amide vibrations (amide I ∼1650 cm^–1^ and amide II ∼1550 cm^–1^), the peak positions were determined by peak fitting using the Fityk software.

#### X-Ray Diffraction Measurements

Small-angle X-ray diffraction (SAXD) measurements were performed at the NCD-SWEET beamline (ALBA Synchrotron, Barcelona, Spain), using a Pilatus 1 M detector with a pixel array of 981 × 1043, each pixel: 172 × 172 μm^2^. The sample-to-detector distance was 2.148 m, and the beam wavelength was 0.999 Å. The temperature for the measurements was 23 °C, and the samples were scanned for 20 s. Silver behenate was used for the calibration of the setup. The one-dimensional SAXD profiles of the scattering intensity as a function of the scattering vector (*q*) were obtained after the integration of the two-dimensional scattering plot, over a 90° segment from the beam center. The scattering vector (*q*) is calculated using the formula: *q* = (4π sin θ)/λ, where θ represents the scattering angle and λ is the wavelength. The positions of the *n*th-order diffraction peak (*q*_*n*_) were determined by peak fitting with the Fityk software, using the Pearson VII function.^60^ Least squares fitting was used to calculate the repeat distance of the lamellar phase (*d*), as *d* = 2*n*π/*q*_*n*_. For peaks that correspond to unknown phases (i.e., not part of a lamellar phase), the spacing at the peak position *q* was calculated as 2π/*q*.

#### Neutron Diffraction Measurements

The neutron diffraction measurements were performed on the LARMOR instrument at ISIS Neutron and Muon Source (Rutherford Appleton Laboratory, UK). The wavelength range of the neutron beam (with a size of 1 × 30 mm) was 1–12.5 Å. The distance between the detector and the sample was 4.4 m. The detector was set at 2θ angle of 5° to the direct beam (area covered 664 × 600 mm; pixel size 4 × 8 mm). The angle of the sample to the beam was 2.5°. An aluminum chamber was used for the sample environment, which allowed a constant temperature of the windows of the chamber at 42 °C to prevent condensation. An empty chamber was used for a background measurement, which was subtracted from each sample. The samples were measured for 4 h each (40 μA/h accelerator proton charge) at 25 °C for each of the three hydration buffer ratios. A direct beam measurement was used for the normalization to the incident flux shape and the detector efficiency.

To monitor the normalized intensity as a function of the scattering vector (*q*), the neutron data were reduced using the Mantid software framework.^61^ The resulting *q*-range was 0.032–0.991 nm^–1^. The Bragg equation was used to convert the scattering angle (2θ) to *q* as *q* = (4π sin θ)/λ. Based on the positions of the equidistant Bragg peaks, the repeat distance (*d*) of the lamellar phase was calculated as *d* = 2*n*π*/q*_*n*_, with *n* representing the diffraction order number of the peak at the position *q*_*n*_.

The intensity of each diffraction order was obtained by fitting the Bragg peaks (Fityk software, with a Pearson VII function).^60^ Next, the structure factor amplitude for each diffraction order (|*F*_*n*_|) was determined using , where *L* is the Lorentz correction factor, which can be assumed equal to *q*, due to the high degree of lipid lamellae orientation. *A*_*n*_, the correction factor for the sample absorption, was calculated with the formula below, where *l* is the thickness of the lipid sample and μ is the linear attenuation coefficient:^62^
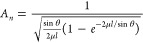


The contrast variation method with D_2_O/H_2_O buffer levels (100%, 50%, and 8%) was used to determine the phase signs of the water profile, which are obtained from the positive or negative signs of the slopes of the linear correlation of the absolute structure factors of the samples hydrated at 100% and 8% D_2_O/H_2_O.^63^ Assuming water is associated with the hydrophilic headgroups located at the boundary of the unit cell, we used the following phase signs’ combination: – + – + for the four diffraction orders detected in the samples. Next, the *F*_*n*_ with the corresponding phase signs is plotted as a function of the D_2_O/H_2_O buffer ratio (Figure S1.2), resulting in a linear fitting for each diffraction order.

The scattering length density (SLD) profile of the SPP profile was obtained by Fourier reconstructions using the structure factor values and the phase signs with the following equation:
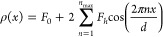
where *x* is the distance in the unit cell, and *x* = 0 represents the center of the unit cell. *F*_0_ represents the scattering density per unit volume. This was calculated using the lipid sample density and its chemical composition (one water molecule per lipid was included).^64^ The SLD profile of the deuterated moiety is determined from the difference between the SLD profile of the deuterated sample and the SLD profile of the protiated sample (both hydrated at 8% D_2_O/H_2_O), and this net SLD profile indicates the location of the deuterated lipid chain.

The SLD data were placed on a “relative absolute” scale using a scaling factor, as described previously.^65−67^ For the SPP NSd7:NP 2:1 sample, in the SLD profile of the NSd7 chain, the peak area (SLD_*a*_) and peak height (SLD_*h*_) were fitted. The peak area obtained from the subtraction of the SLD profiles of the SPP NS:NP 2:1 sample from the SPP NSd7:NP 2:1 sample represents the scattering of the deuterium atoms from the CER NS sphingosine chain (SLD_dif_). The relative absolute SLD profile (SLD_correct_) was calculated as follows:
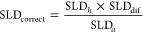


The scaling factor that was then applied to the structure factor values (*F*_*n*_) was calculated as the ratio between the SLD_correct_ and SLD_*h*_ values.

### Simulation Methods

#### Simulation Procedures

CG simulations were performed using models for CER NS, CER NP, CHOL, FFA C24, and water developed using the multistate iterative Boltzmann inversion (MS-IBI) method in earlier work.^54,59,68−74^ Like the experiments, CER acyl and sphingoid base chains were 24 and 18 carbon atoms, respectively. The lipid compositions studied matched those in Table 1 for SPP NS:NP 1:2 and 2:1 except for CHOL, which was half of the amount used in the experiments (i.e., the molar ratio of CER:CHOL:FFA was 1:0.5:1). Based on prior experimental work, this is approximately the largest amount of CHOL in an equimolar mixture of CERs with an acyl chain length of C24 and FFA C24 that exclusively forms the SPP without phase-separated crystalline CHOL.^41,43,45,58,75^ Thus, the experimental SPP contains less than an equimolar amount of CHOL and so the simulated systems at the molar ratio of 1:0.5:1 most closely represent the SPP.

All simulated systems were initialized with 2200 lipid molecules placed randomly between two layers of 11 000 CG water beads located on the top and bottom of the simulation box. The total of 22 000 CG water beads corresponds to 88 000 water molecules, and thus, 40 water molecules/lipid. The CG lipids were then self-assembled into a multilayer stack of three bilayers (six leaflets), as illustrated in Figure S3.1, over the course of 1–2 μs of simulation using both temperature and shape annealing (i.e., expanding and contracting the simulation box) as described in Section S2. A three-bilayer stack was simulated as it more closely mimics the experimental SC models. Additionally, it was shown by Shamaprasad et al.^54^ that the number of leaflets included in the membrane model (2, 4, or 6) affects structural properties, such as bilayer thickness and hydrogen bonding. Interlayer hydrogen bonding is observed experimentally^76^ and seen between the inner leaflets of three-bilayer systems^54^ but not observed in single-bilayer systems, where the hydrogen bonding is dominated by interactions with water.

The final configuration from the self-assembled CG simulation was converted to an equivalent atomistic configuration using the reverse-mapping procedure similar to that proposed in the study by Shamaprasad et al.^54^ In this approach, the composition and in-plane morphology of each leaflet is preserved along with the hairpin or linear conformations of the CERs (i.e., the sphingosine and acyl tails point in the same direction or opposite directions) and the average lipid tilt angle. Some CG water beads were self-assembled into the inner four leaflets of the three-bilayer stack (i.e., the leaflets that did not contact bulk water; Figure S3.1). These water beads were included in the reverse-mapped atomistic system. This differs from Shamaprasad et al.’s study in which the reverse-mapping procedure was expedited by omitting CG water beads in the inner leaflets.^54^ Here, four atomistic water molecules were placed randomly with the same center-of-mass position as the CG water bead they replaced. Infrequently, a water molecule had contact with another molecule and was shifted 1.5 Å in the *x*-direction to avoid high energy overlaps.

The reverse-mapped atomistic configurations were equilibrated using atomistic simulations. CHARMM36-based atomistic models for the CERs and FFA^53,54,59,77^ were combined with the TIP3P water model^78^ and an atomistic CHOL model.^79^ To relax the reverse-mapped configuration, a short atomistic simulation was performed using the GROMACS 2020.6 simulation engine, employing a 1 fs time step.^80^ This strategy is based on the reasonable assumption that the starting CG conformation is locally relaxed, given that the CG multilayers have already undergone self-assembly and were run for several hundred nanoseconds resulting in a stable, unchanging structure. The simulations began with an energy minimization phase employing the steepest descent algorithm for a maximum of 200 000 simulation steps to resolve any lipid overlaps. Subsequently, the lipids underwent a brief equilibration process beginning in the constant-pressure, constant-temperature (*NPT*) ensemble, utilizing the Nosé–Hoover thermostat^81^ and Parrinello–Rahman barostat^82^ that lasted 25 ns at 305 K and 1 bar, with semi-isotropic pressure control. The final 2 ns of the NPT equilibration was deemed the production run at 305 K (32 °C) and 1 bar.

Three independent and complete simulations, including the CG self-assembly, reverse-mapping to the atomistic configuration, and atomistic simulation, were performed for the SPP NS:NP 1:2 and SPP NS:NP 2:1 models. All topology manipulations (i.e., creation of the CG initial structures and the reverse-mapping process) utilized the MoSDeF software library, mBuild.^83−86^ Unless specified differently, results from each simulation replicate are the average of the 200 frames of data collected in the last 2 ns of the atomistic simulations. Results are reported as the mean and standard deviation of the average values from the three replicated simulations. Analyses of statistically significantly larger values were performed using the unpaired *t* test with the one-tailed significance level set at *p* < 0.05 or *p* < 0.07. Differences in mean values were considered significant when *p* < 0.05 for two tails.

#### Analysis of Simulated Results

Several structural properties were calculated for the self-assembled three-bilayer stack. These include the area per lipid (APL), bilayer thickness, nematic order parameter (S_2_), and the lipid tilt angle; all were calculated for leaflets that do not contact bulk water (Figure S3.1). APL was calculated by dividing the cross-sectional area of the simulation box by the average number of lipid headgroups per leaflet in the four inner leaflets. All other structural properties were determined for the central bilayer (i.e., the middle two leaflets; Figure S3.1). The bilayer thickness was calculated from the distance between the two peaks in the mass density profile representing the headgroup regions of the central bilayer in the three-bilayer stack. The lipid tilt angle was calculated by measuring the angle between the vector between the tail director and the *z*-axis. The director vector is an eigenvector related to the minimum eigenvalue of the inertia tensor. The S_2_-order parameter was calculated by taking the largest eigenvalue of the nematic tensor as described in the Supporting Information of the study by Wilson.^87^ The analysis of the lipid systems was performed utilizing the MDTraj^88^ and SciPy^89^ software packages. Additional details about the structural property calculations are provided in Section S3.

The simulated SLD profile was calculated by weighting the composition histogram along the *z*-dimension by the atomic neutron scattering lengths. The histograms were generated using a histogram bin size of 0.5 Å and averaged over the last 200 simulation frames. The protiated profile contains only lipids (no water). The simulated SLD profile for the system containing NSd7 was created by replacing the hydrogens on the last three alkyl carbons on the sphingosine chain with deuterium in the 200 frames of data collected in the simulations (Figure S1.1). From there, the curve of the difference between the deuterated and protiated curves was calculated, symmetrized, and scaled such that the minimum and maximum values were at 0 and 100, respectively. For comparisons with the experimental curve, the calculated difference between the experimental protiated and deuterated structure factor data hydrated at 8% D_2_O/H_2_O was scaled between 0 and 100 for the simulated SLD curve.

Intermolecular hydrogen bond interactions were calculated from the reverse-mapped atomistic models using the GROMACS 2020.6^80^*hbond* module. A hydrogen bond was formed when the distance between the donor and acceptor is less than 0.35 nm and the angle formed by the donor, acceptor, and hydrogen is less than 30°. Intramolecular hydrogen bonds were not considered because these are unlikely given the positions of the hydrogen bonding sites and limited CER flexibility in this region. Hydroxyl and amine groups are identified as donors, while oxygen and nitrogen atoms are recognized as acceptors. Notably, CERs can function as both hydrogen bond donors and acceptors. Altogether, the CERs, CHOL, and FFA molecules include eight hydrogen bonding sites, which can form 33 different hydrogen bonding pairs (Section S4). Hydrogen bonds with the carbonyl (C=O) groups and with the amine group (N–H) in the CER NS and CER NP molecules were assumed to be related to the observed shifts in the amide I and amide II FTIR spectrum, respectively. The hydrogen bonds were calculated for each atom in each frame and then averaged across the 200 frames and averaged over the three independent simulations. To focus on hydrogen bond formation that is most comparable to those occurring in the experiments, only headgroup regions within the four inner leaflets of the three-bilayer-simulated membrane were considered. This allows for the study of lipid–lipid hydrogen bonding without the influence of large amounts of water on the outer leaflets. More detailed information and schematics of the atoms participating in hydrogen bonding can be found in Section S4.

## Results and Discussion

### Lamellar and Lateral Organization of the Two Lipid Models

**Figure 1:**
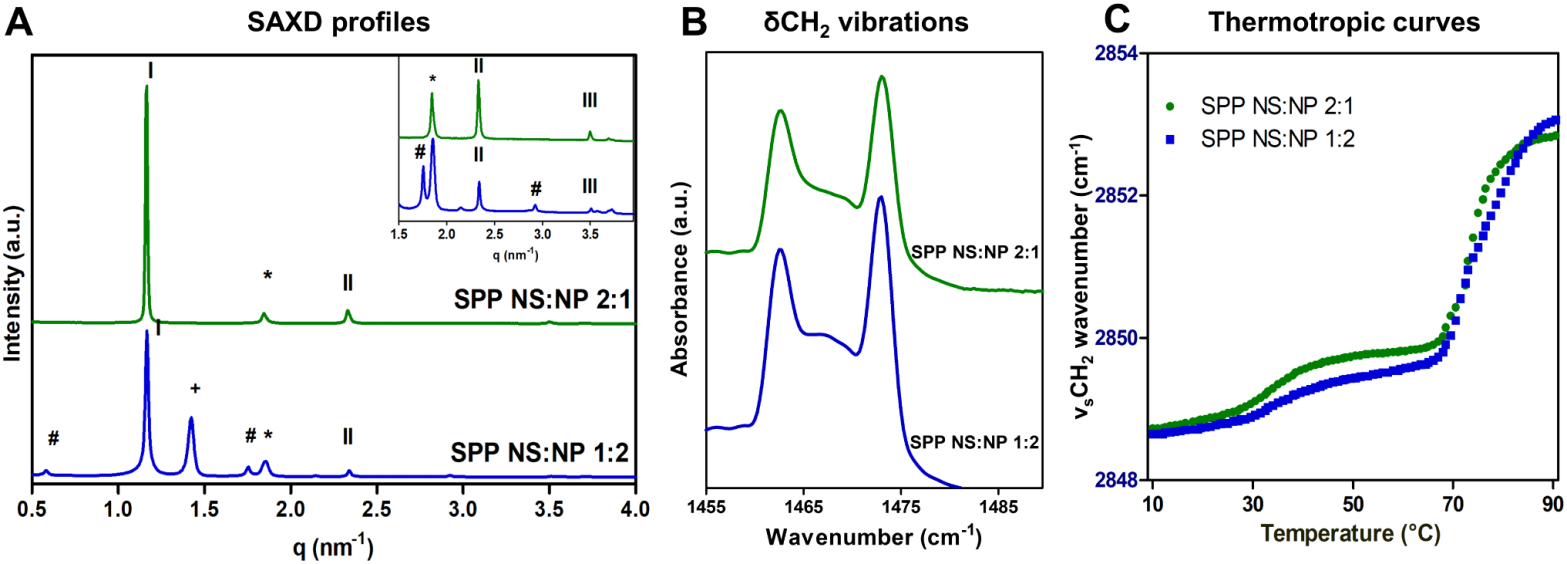
(A) SAXD profiles of the SPP NS:NP 1:2 (blue, lower) and SPP NS:NP 2:1 (green, upper) models. The SPP diffraction orders are indicated with Roman numbers, the asterisk (*) indicates phase-separated CHOL peaks, the hash (#) indicates the peaks corresponding to a lamellar phase with a *d*-spacing of 10.8 nm, and the plus (+) indicates an unknown peak. The top-right panel shows an expanded view of the profile for the *q*-range of 1.5–3.6 nm^–1^. (B) δCH_2_ vibrations of the two protiated models, measured at 10 °C. (C) Thermotropic curves of the SPP NS:NP 1:2 and 2:1 models, showing the ν_s_CH_2_ wavenumbers as a function of temperature in the range of 10–90 °C.

The lamellar organization of the two lipid models with the CER NS:CER NP ratio of 1:2 and 2:1 was examined with SAXD. Figure 1A shows the diffraction profiles of the two systems. The SPP NS:NP 2:1 system is characterized by a series of equidistant peaks, indicating a lamellar phase with a repeat distance of 5.4 nm, the SPP. The only peak that is not assigned to the SPP is attributed to phase-separated crystalline CHOL (positioned at *q* = 1.8 nm^–1^). The diffraction profile of the SPP NS:NP 1:2 model also shows a series of three equidistant peaks attributed to a lamellar phase with a repeat distance of 5.4 nm, indicating the formation of the SPP. However, the SAXD profile of this model also shows two other phases. The hash symbols designate a lamellar phase with a *d*-spacing twice that of the SPP (*d* = 10.8 nm, first diffraction peak at *q* = 0.58 nm^–1^), which has its other diffraction orders overlapping the first, second, and third SPP peaks. There are previous reports about a lamellar phase with ∼10.6 nm repeat distance in lipid compositions that included CER NH C24,^47,48^ CER NS C24,^58,90^ and a mixture of CER NS C24/CER NH C24.^91^ This phase with a suggested double-bilayer structure was first reported in 1993 but was not considered representative for SC and was suggested to be an artifact of the sample preparation technique used.^92^

The peak indicated by the plus symbol in Figure 1A (SPP NS:NP 1:2 model) identifies an unknown phase with a peak at *q* = 1.4 nm^–1^ (spacing 4.4 nm), which has been observed previously in other compositions and suggested to be a crystalline phase containing CER NP with an acyl chain of 24 carbon atoms.^93^ Dahlen et al. reported that pure CER NP can adopt a V-shape conformation with a tilt angle of ∼41°. A spacing of 4.4 nm was also reported in an equimolar model of CER NP C24, CHOL, and FFA C24, and the 4.4 nm phase was suggested to represent the V-shape arrangement of CER NP.^50^

Unlike the denoted phase separation for the SPP NS:NP 1:2 model, lipid models forming the LPP (the same ratio between the lipid, but with the addition of CER EOS) did not form multiphase systems, even when the CER NS:CER NP molar ratio was 1:2.^34^ This suggests that the addition of CER EOS improves the miscibility of the lipids in the model.^41,94^

The CER NS:CER NP ratio is different in human SC, compared to murine SC. The most abundant CER subclass in murine SC is CER NS, while in human SC, one of the most abundant CER subclasses is CER NP.^8^ Previous studies showed that even though the CER composition is different, the lamellar organization is similar in human and murine SC.^14^

Next, the lateral lipid organization was examined using FTIR. The δCH_2_ vibrations for the protiated SPP NS:NP 1:2 and SPP NS:NP 2:1 models show two clear peaks at approximately 1462 and 1473 cm^–1^ (characteristic for orthorhombic packing) and a smaller central peak at 1467 cm^–1^ (attributed to hexagonal lipid packing) (Figure 1B). Peak fitting with Python scripts was used to determine accurately the δCH_2_ peak position, the δCH_2_ peak splitting (distance between the two peaks caused by an orthorhombic packing), and then the peak height ratio of the average of the two orthorhombic peaks and the hexagonal middle peak (OR/MID). While the δCH_2_ peak splitting distance was the same for both models (10.3 ± 0.08 cm^–1^, Table 2), the SPP NS:NP 1:2 model had a significantly higher central peak relative to the two orthorhombic peaks, and thus a lower OR/MID ratio (1.9 ± 0.1), than the SPP NS:NP 2:1 model (OR/MID ratio 2.3 ± 0.1). The δCH_2_ vibrations suggest that both models adopt primarily an orthorhombic packing; however, a small fraction of lipids forms a hexagonal packing, which is higher in the SPP NS:NP 1:2 model. In Figure S1.3, the δCH_2_ vibrations are provided in the 10–50 °C temperature range. These vibrations indicate an orthorhombic to hexagonal phase transition.

**Table 2:** δCH_2_ Peak Splitting Distance, δCH_2_ Peak Height Ratio (OR/MID) of the two SPP Models, at 10°C, and the Mid-Phase Transition Temperatures (*T*_m_O-H and *T*_m_H-L)^a^

Lipid model	δCH_2_ peak splitting ± SD	OR/MID peak height ratio ± SD	*T*_m_O–H ± SD (°C)	*T*_m_H-L ± SD (°C)
SPP NS:NP 1:2	10.3 ± 0.1	1.9 ± 0.1	32.4 ± 0.7	74.6 ± 0.4
SPP NS:NP 2:1	10.3 ± 0.1	2.3 ± 0.1	32.6 ± 1.6	70.8 ± 1.8

a Data are shown as an average of three measurements for each composition ± SD.

The thermotropic curves of the ν_s_CH_2_ vibrations are shown in Figure 1C. At 10 °C, the ν_s_CH_2_ wavenumber is <2849 cm^–1^ indicating a high conformational order, while at 32 °C, there is a transition to a less ordered system, indicated by the increase of the ν_s_CH_2_ wavenumber. Correlating the ν_s_CH_2_ vibrations with the observations from the δCH_2_ vibrations (Figure S1.3), this corresponds to the transition from the orthorhombic to hexagonal lipid packing. The mid-phase transition temperatures were similar for the two models, as indicated in Table 2. When the temperature is further increased, another transition can be observed at ∼70 °C as depicted in Figure 1C, from hexagonal lipid packing to a liquid phase. The difference between the mid-phase transition temperatures (*T*_m_H-L) of the two models is statistically significant (*p* < 0.05), as the SPP NS:NP 2:1 model showed a sharper transition with *T*_m_H-L = 70.8 ± 1.8 °C than the transition observed for the SPP NS:NP 1:2 model with *T*_m_H-L = 74.6 ± 0.4 °C (Table 2).

Structural properties derived from simulations of the two SPP lipid models can provide insights into their lamellar and lateral organization. Properties calculated for leaflets without bulk water contact (Figure S3.1), either the central bilayer (for bilayer thickness, lipid tilt angle, and nematic order parameter (S_2_)) or the inner four leaflets (for APL), are summarized in Table S3.1. Bilayer thicknesses for the two SPP models are found to be the same (∼5.35 nm) and similar to the 5.4 nm SPP repeat distance observed in the SAXD measurements for both SPP models. Likewise, the APL (0.334 nm^2^), tilt angle (∼12°), and S_2_ (0.92) are the same for the two models, and the S_2_ values are consistent with a well-ordered, hexagonal or orthorhombic, lamellar system, observed in a previous study of an SC lipid model.^58^ Together, these results indicate, as was observed in the experiments, that the lamellar and lateral organization of the SPP phases are unaffected by changing the CER headgroup from CER NS to CER NP.

Phase separation, such as that detected by SAXD for the SPP NS:NP 1:2 and 2:1 models, is not observed in the simulations due to the size of the systems studied. As a result, the composition of the simulated lamellar system can differ from that in the experimental lamellar phase whenever other phases are also present experimentally. For example, phase-separated crystalline CHOL in the experimental systems of this study reduces the CHOL:CER molar ratio in the SPP to less than one and is accounted for in the simulations by studying a 0.5 rather than 1 molar ratio for CHOL. If some of the CER NP in the experimental SPP NS:NP 1:2 model is phase-separated, this would also cause a lower presence of CER NP in the SPP of this model compared with the nominal composition. And, as a result, the CER NP composition used in the simulations of the SPP NS:NP 1:2 model would be higher than in the experiments. Whether any CER NP has phase-separated, the amount and its effect, if any, on the simulation results is unknown and reserved for a future study.

### Thermotropic Behavior Indicates Phase Separation in SPP NS:NP 1:2 Model

**Figure 2:**
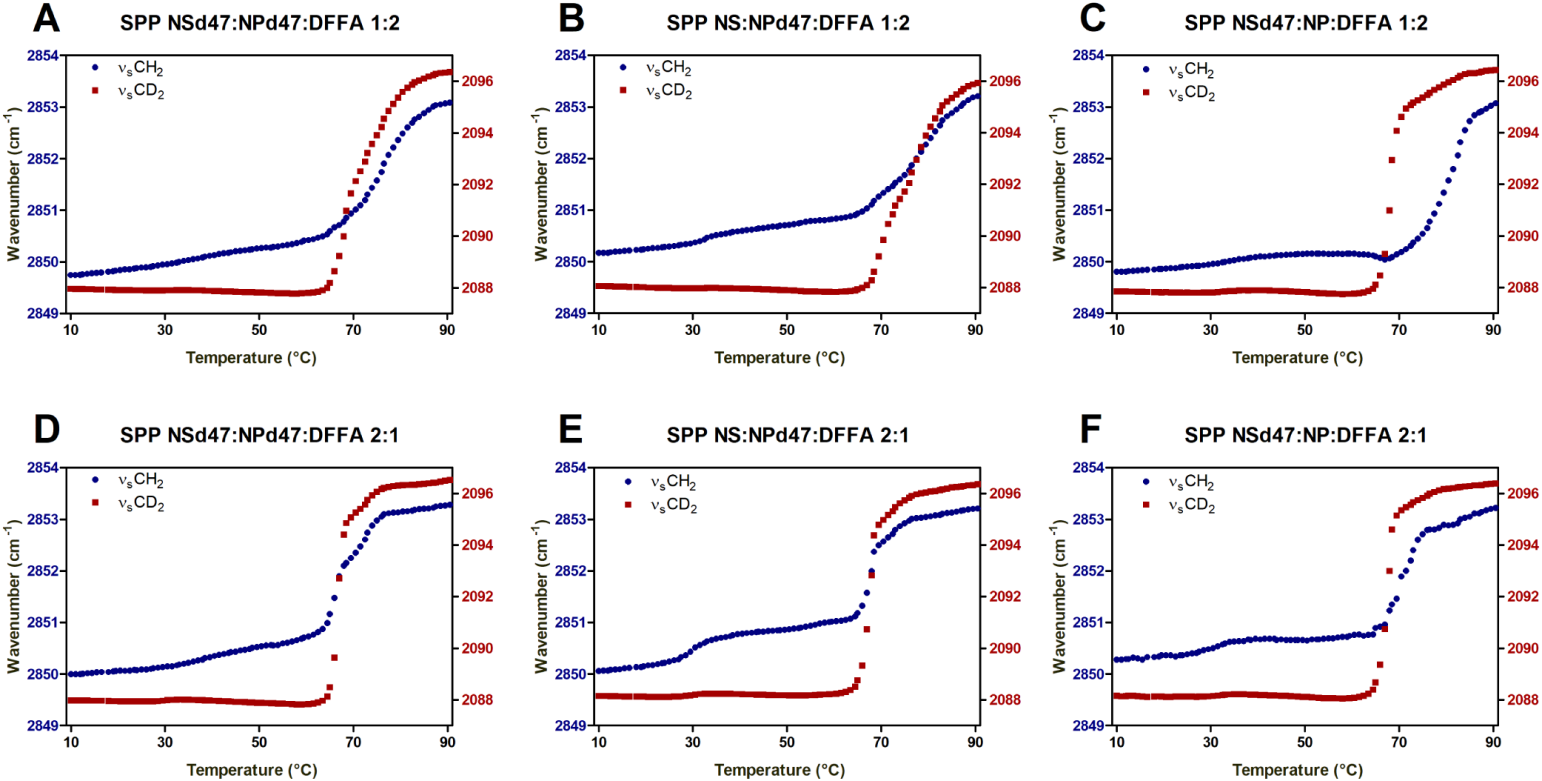
Thermotropic curves of the ν_s_CH_2_ and ν_s_CD_2_ vibrations for the SPP NS:NP 1:2 (A–C) and 2:1 (D–F) ratio models with different deuterated chains. The wavenumbers of the ν_s_CH_2_ and ν_s_CD_2_ peak positions (left and right *y*-axes, respectively) are plotted in the 10–90 °C temperature range. Data are shown as an average of three measurements for each composition.

To further investigate the lipid mixing of the models, some of the lipids were replaced with their deuterated counterparts: perdeuterated acyl chain of CER NP and/or CER NS and perdeuterated FFA C24 (Table 1). The thermotropic curves of these models (Figure 2) show different behavior of the two protiated SPP models (1:2 and 2:1 ratios). The SPP NS:NP 2:1 models are characterized by sharp transitions from hexagonal to liquid phase, similar to the protiated sample discussed previously (Figure 1C). Moreover, a similar thermotropic response of the ν_s_CH_2_ and ν_s_CD_2_ vibrations is detected for the SPP NS:NP 2:1 models. This indicates that the hexagonal to liquid phase transitions of the protiated and deuterated lipids occur in the same temperature range; thus, this transition does not indicate phase separation of the lipids.

Figure 2 shows that the SPP NS:NP 1:2 models have a larger hexagonal to liquid phase transition temperature range than the SPP NS:NP 2:1 model. On average, this phase transition of the SPP NS:NP 1:2 models occurs over a 20 °C temperature range, while for the SPP NS:NP 2:1 models, the range is 12 °C. However, the protiated and deuterated chains of both SPP NSd47:NPd47:DFFA 1:2 and SPP NS:NPd47:DFFA 1:2 models melt in the same temperature range (Figure 2A,B). The SPP NSd47:NP:DFFA 1:2 model (Figure 2C) shows different ranges in transition temperature for the deuterated and protiated lipids, indicating that lipid domains of different compositions are formed in this model.

The mid-transition temperature of the deuterated and protiated chains in the SPP NSd47:NP DFFA 1:2 deuterated models is significantly different, as shown in Table 3. This suggests that the lipids in this mixture do not mix homogeneously. The SAXD data of the SPP NS:NP 1:2 model showed an unidentified phase with a spacing at 4.4 nm, which as discussed above might contain primarily CER NP. The differences in the mid-transition temperatures between the protiated and deuterated lipids could be caused by this crystalline CER NP-rich phase. Due to the presence of a high concentration of CER NP, it is expected to have a delayed onset of the melting process of the protiated chains. This is in agreement with the observation that in the SPP NSd47:NPd47:DFFA 1:2 model, the protiated chains do not show a delay in the hexagonal liquid transition (Figure 2D).

**Table 3:** Mid-Phase Transition Temperature of the Hexagonal–Liquid Phase (*T*_m_H-L), the δCD_2_ Peak Splitting, and the OR/MID δCD_2_ Peak Height Ratio of the Deuterated SPP Models^a^

Lipid model	*T*_m_H-L ± SD (°C)	δCD_2_ peak splitting ± SD(cm^–1^)	OR/MID peak height ratio ± SD
SPP NSd47:NPd47:DFFA 1:2	75.3 ± 0.6	7.2 ± 0.1	5.3 ± 0.3
SPP NSd47:NPd47:DFFA 2:1	68.6 ± 0.6	7.2 ± 0.1	5.3 ± 0.4
SPP NS:NPd47:DFFA 1:2	76.8 ± 0.4	5.9 ± 0.1	3.3 ± 0.2
SPP NS:NPd47:DFFA 2:1	68.1 ± 0.9	5.0 ± 0.1	2.9 ± 0.2
SPP NSd47:NP:DFFA 1:2	80.7 ± 1.4	6.4 ± 0.1	2.7 ± 0.2
SPP NSd47:NP:DFFA 2:1	74.5 ± 0.9	6.2 ± 0.1	3.4 ± 0.2

a The scissoring peak data are calculated at 10 °C. Data represent an average of three measurements for each composition with the standard deviations.

There is only a weak phase transition from orthorhombic to hexagonal packing of the protiated lipid chains in most compositions shown in Figure 2, as in most compositions, the ν_s_CH_2_ wavenumber shows a slight and steady increase up to 50 °C. This is an indication that the protiated lipids (CHOL, sphingosine, and phytosphingosine chains) adopt primarily a hexagonal organization (except in the SPP NS:NPd47:DFFA 2:1 model). Unlike these SPP systems, in the LPP models studied recently, clear transitions from orthorhombic to hexagonal phases were noticed in the thermotropic plots of the deuterated samples.^34^ It has been previously reported that CER EOS may enhance the formation of the orthorhombic phase, as the long acyl chains of CER EOS might increase the van der Waals interactions.^41,95^ Thus, the presence of CER EOS acyl chains could be a possible explanation for the aforementioned differences between these SPP systems and the LPP models.

At 32 °C (skin temperature), the wavenumbers of the ν_s_CH_2_ vibration in the SPP NSd47:NPd47:DFFA 1:2 and 2:1 systems are 2850.1 ± 0.2 cm^–1^ and 2850.2 ± 0.1 cm^–1^, respectively. The stretching wavenumber at this temperature indicates that the protiated sphingosine and phytosphingosine chains and CHOL have less conformational ordering than at 10 °C. The conformational disordering of the sphingosine chain of CER NS was previously reported by Engberg et al.^45^ who labeled this phase as a fluid, highly mobile phase, based on the ^2^H NMR results. However, a clear distinction should be made regarding the packing of the sphingosine chain of CER NS, as in FTIR terminology, a fluid disordered phase is characterized by a ν_s_CH_2_ wavenumber >2853 cm^–1^ and a ν_s_CD_2_ wavenumber >2096 cm^–1^. In Engberg et al.’s study, the ν_s_CH_2_ wavenumber of the deuterated sphingosine CER NS chain is ∼2089.5 cm^–1^,^45^ which indicates some conformational disordering but not a fluid phase as detected by FTIR.

### The Linear Conformation of CER NS and CER NP Is Similar to LPP Models

**Figure 3:**
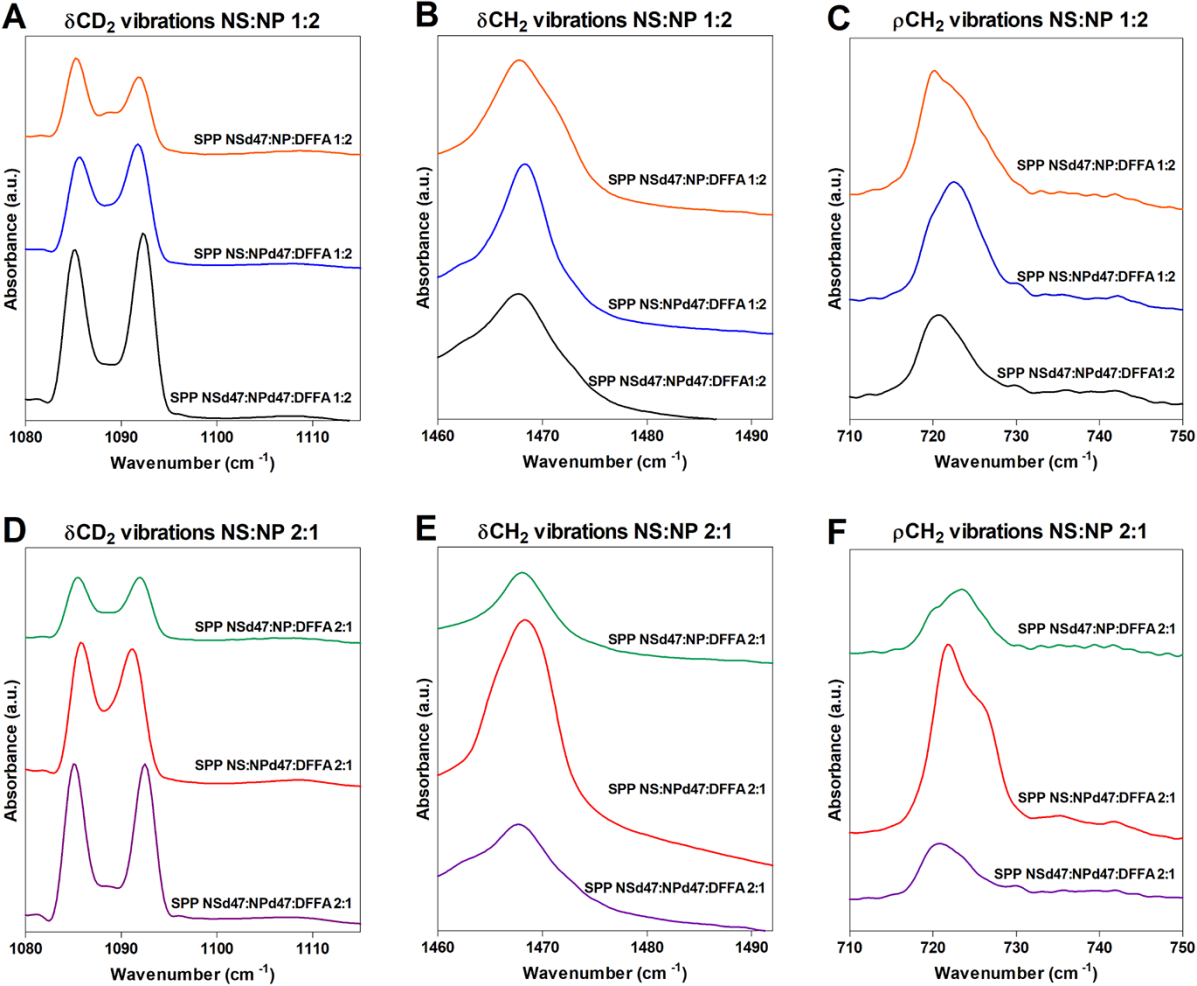
δCD_2_ vibrations (A,D), δCH_2_ vibrations (B,E), and ρCH_2_ vibrations (C,F) for the partially deuterated models SPP NSd47:NP:DFFA, SPP NS:NPd47:DFFA, and SPP NSd47:NPd47:DFFA with the CER NS:CER NP ratios of 1:2 (top row of panels) and 2:1 (bottom row), measured at 10 °C.

The mixing of the lipid chains is further examined using the scissoring vibrations. The results are provided in Figure 3, which shows the splitting of the δCD_2_ and δCH_2_ vibrations in the FTIR spectra of the various compositions. In an orthorhombic packing, the hydrocarbon lipid chains are packed tightly, allowing short-range coupling of the CH_2_–CH_2_ groups, resulting in a peak splitting of the δCH_2_ vibrations. Similarly, when deuterated lipid chains are included in the models, the CD_2_–CD_2_ chains interact if they are neighboring, resulting in two separated δCD_2_ peaks at ∼1085 and ∼1092 cm^–1^. The size of the orthorhombic domains determines the δCD_2_ peak splitting distance, with a maximum peak splitting distance of 7.3 ± 0.1 cm^–1^, obtained when the lipid domains are around 100 chains.^96,97^ However, if the deuterated chains are neighboring protiated chains that participate in the same lattice, CD_2_–CH_2_ interactions occur, resulting in the loss of the CD_2_–CD_2_ chain frequency coupling. A central peak is formed in both the δCD_2_ vibration (∼1088 cm^–1^) and δCH_2_ vibration (∼1468 cm^–1^), resulting in a shallower depth between the two orthorhombic peaks.

Large deuterated domains are formed in the SPP NSd47:NPd47:DFFA models (1:2 and 2:1 ratios; Figure 3A,D), as the distance of the δCD_2_ peak splitting is 7.2 ± 0.1 cm^–1^ in both compositions (Table 3). These values are close to the maximum δCD_2_ peak splitting value obtained for pure DFFA C24, which suggests that the acyl chains of CER NS and CER NP are neighboring the DFFA chains. An indication of the number of CH_2_–CD_2_ interactions is the peak ratio of the average peak height of the two orthorhombic peaks and the peak height of the central peak (OR/MID peak height ratio). This ratio was calculated by peak fitting. The OR/MID ratio values obtained for this model are very high, indicating that the CD_2_–CD_2_ chain interactions are predominant in the system (Table 3).

To further investigate the deuterated lipid domains in the SPP models, CER NSd47 was replaced by the protiated CER NS in the SPP NS:NPd47:DFFA (1:2 and 2:1) models. Both models indicate that smaller deuterated lipid domains are present (Table 3 and Figure 3). The OR/MID peak height ratios of these models are significantly decreased compared to the SPP NSd47:NPd47:DFFA models, indicating that in the SPP NS:NPd47:DFFA models, there are significantly less CD_2_–CD_2_ chain interactions and more CH_2_–CD_2_ interactions. This confirms that the deuterated acyl chain of CER NS is part of the deuterated domains formed in the SPP NSd47:NPd47:DFFA models. Neighboring of these chains is only possible when CER NS and CER NP are present in a linear conformation in the SPP models, with the acyl chain and phytosphingosine chain on either side of the headgroup. The difference in the δCD_2_ peak splitting values between the SPP NS:NPd47:DFFA 1:2 and SPP NS:NPd47:DFFA 2:1 models (Table 3) is likely also caused by the different concentrations of the deuterated CER NP in the models. Next, CER NPd47 was replaced by its protiated counterpart resulting in the SPP NSd47:NP:DFFA 1:2 and 2:1 models. The conclusions of the SPP NS:NPd47:DFFA models also apply to the SPP NSd47:NP:DFFA models: smaller deuterated lipid domains than the SPP NSd47:NPd47:DFFA models and a linear arrangement of CER NS and CER NP.

The linear conformations of CER NS and CER NP are also observed in LPP models with the same composition but including CER EOS (CER EOS: CER NS: CER NP: CHOL: FFA C24).^34,63^ Studies of the LPP models using neutron diffraction and the peak splitting observed in FTIR measurements both indicate that the acyl chains of CER NS and CER NP are neighboring FFA C24, regardless of the CER NS:CER NP molar ratio, similar to the results observed in the SPP model in the present study. The linear conformation of CER NS was reported in previous studies using LPP models with different compositions^98,99^ and SPP models;^43,45,58^ however, the conformation of CER NP in SPP models was often debated, with different possible arrangements suggested, such as V-shape^50,93^ or hairpin.^51^ The V-shape configuration was detected in a phase with a repeat distance of 4.3 nm, likely representing phase-separated CER NP. In the SPP with a 5.4 nm repeat distance, CER NP is suggested to be arranged in either hairpin or linear conformations.

When CER NS and CER NP are in a linear conformation, the acyl chains of CER NS and CER NP are neighboring the FFAs, as discussed above, consequently, the sphingosine and phytosphingosine chains of the CERs and CHOL are also neighboring in another part of the repeating unit of the SPP. The δCH_2_ vibrations of the deuterated SPP models (SPP NSd47:NPd47:DFFA, SPP NSd47:NP:DFFA, and SPP NS:NPd47:DFFA 1:2 and 2:1 ratios) are characterized by the presence of a singlet at 1468 cm^–1^ (Figure 3B,E). As the sphingosine chains and CHOL are neighboring, an absence of the doublet in the δCH_2_ vibrations indicates that there is almost no orthorhombic packing of these protiated lipid chains. To investigate this further, the rocking vibrations are also examined (Figure 3C,F): a broad single peak is observed at ∼720 cm^–1^ for the SPP NSd47:NPd47:DFFA models (1:2 and 2:1 ratios), as well. The singlet in these two models suggests that the neighboring phytosphingosine chains and the CHOL are mainly hexagonally packed.

Within the simulations, only ∼35% of the CERs in the four inner leaflets are found to be in the linear conformation (Table S3.2), which is less than is observed in the experiments presented herein. The fraction of linear CERs is the same for CERs NS and NP in both the simulated SPP NS:NP 1:2 and SPP NS:NP 2:1 models. This fraction of linear CERs is also consistent with CG self-assembled three-bilayer models for other systems containing CERs, CHOL, and FFA.^54^ Potential causes for fewer linear CERs in the simulations are being explored including the possibility of modifying the CER CG model. While the model allows for the self-assembly of multilayers, and as such avoids the biases present in preassembled systems (i.e., the starting CER conformation dictates its final conformation), further model refinement may be necessary to increase the fraction of CERs adopting the linear conformation, which will be the subject of future study.

### Neutron Diffraction Shows the Symmetric Structure of the SPP

**Figure 4:**
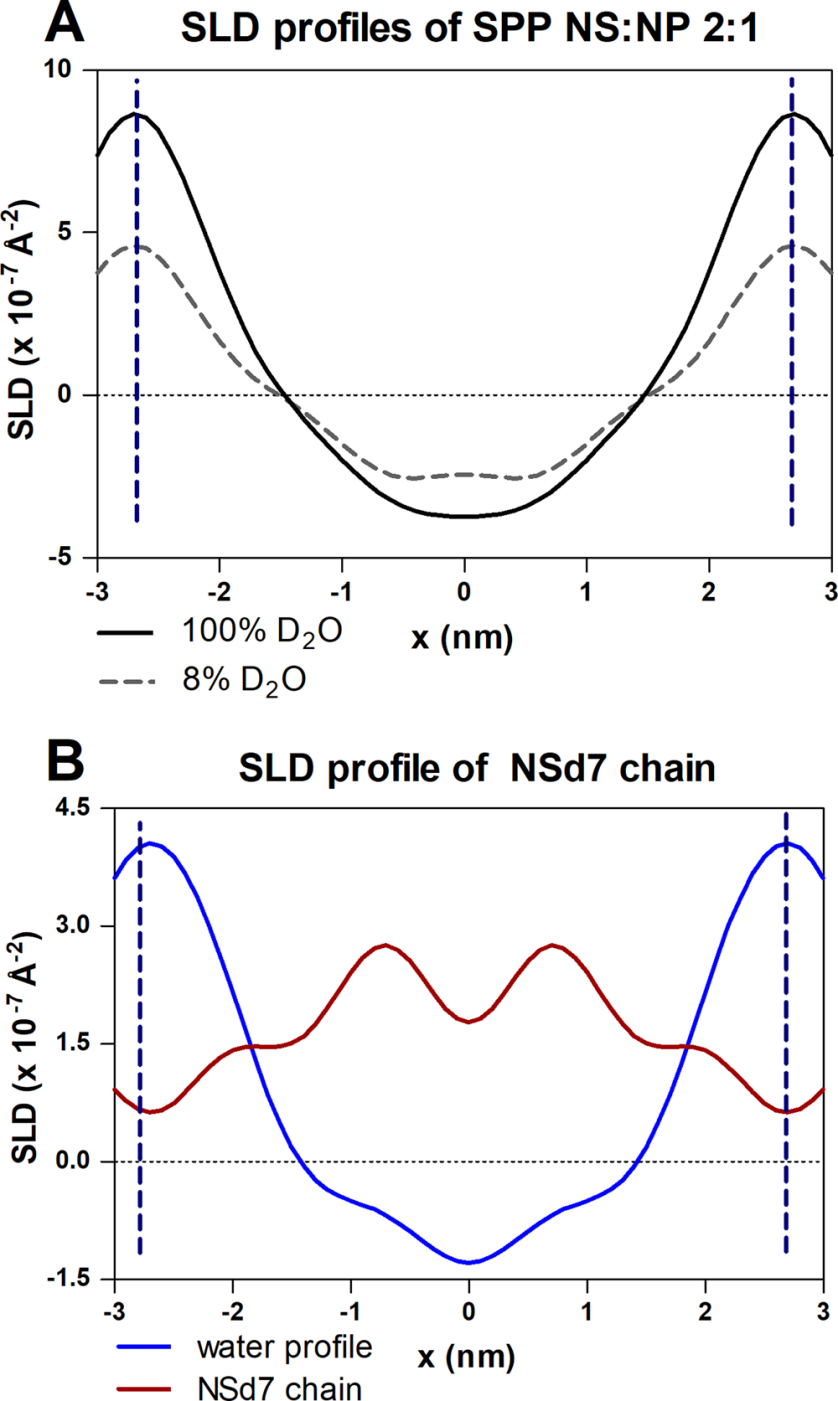
(A) SLD profile of the SPP NS:NP 2:1 sample hydrated at 100% and 8% D_2_O/H_2_O buffer and (B) SLD water profile (in blue) of the sample and the SLD profile of the CER NSd7 chain (in red). The vertical dashed lines indicate the borders of the repeating unit of the SPP.

For the neutron diffraction measurements, the SPP NS:NP 2:1 model was selected to avoid overlap of the diffraction peaks with peaks from the unknown phases observed in the SPP NS:NP 1:2 model. The SLD profiles of the protiated SPP NS:NP 2:1 sample, hydrated at 8% and 100% D_2_O, are shown in Figure 4A. The profile of the protiated sample is characterized by a high SLD value at the borders of the unit cells, indicating that the lipid headgroups are located at the boundary of the unit cell.

The SLD profile of the CER NSd7 chain (in the SPP NSd7:NP 2:1 sample) displays two peaks at the position of ∼0.7 nm from the unit cell center (∼2 nm from the unit cell border) (Figure 4B). A linearly extended sphingosine chain of C18 chain length corresponds to a length of ∼1.9 nm, assuming a C–C bond length of 0.125 nm and 15 C–C bonds.^99^ Thus, the SLD profile intensity shows the location of the NSd7 terminally deuterated chain with the CER headgroup at the unit cell border. The proposed arrangement is schematically shown in Figure S1.4.

The neutron diffraction data indicate a symmetric structure of the SPP unit, also for the CER NSd7 chain, in which the end of the sphingosine chain is located symmetrically at a distance of ∼0.7 nm on both sides of the center of the unit cell. This contrasts with the FTIR results that suggest an asymmetric arrangement, including for CER NS. The FTIR results of the SPP NS:NP 2:1 system indicate that the acyl chains of CER NS, CER NP, and FFA C24 are neighboring, as shown by the large lipid domains described by the scissoring vibration. This suggests an asymmetric arrangement in the SPP profile: on one side of the unit cell, the CHOL neighbors the phytosphingosine chains, while the FFAs are positioned next to the acyl chains of the CERs on the other side. However, while the FTIR scissoring vibrations provide information about the domain sizes, neutron diffraction shows the overall average orientation of the lipids in the system. If the asymmetric arrangement is present in two mirrored orientations with lipid domains larger than 100 chains, FTIR will detect an asymmetric arrangement (based on the interactions between neighboring lipid chains), while neutron diffraction will detect this as a symmetric arrangement, as this technique provides information based on the sum of the two stacks of mirror arrangements (Figure S1.4).

This study shows for the first time in the same SPP composition that the neutron diffraction data indicate a symmetric arrangement, while the FTIR data can only be explained by an asymmetric arrangement. Engberg et al. already proposed an asymmetric arrangement solely based on the FTIR data for a model containing CER NS.^45^

**Figure 5:**
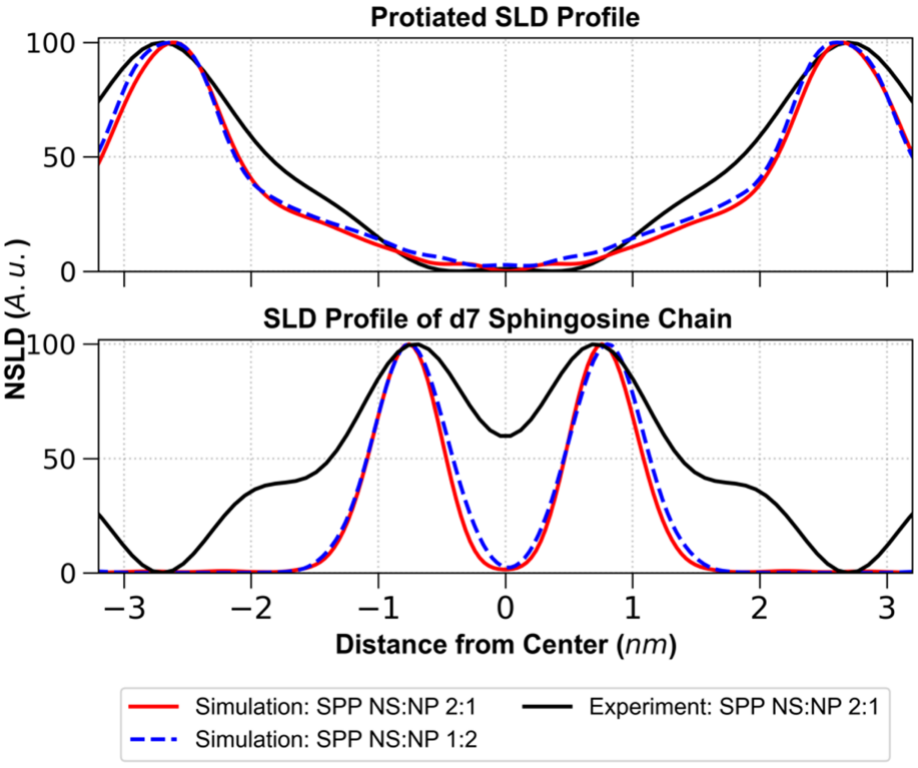
Top: SLD profile of the SPP NS:NP 2:1 protiated sample hydrated at 8% D_2_O/H_2_O buffer (black) compared with SLD profiles of the central bilayer of the three-bilayer stack from one simulation each of the SPP NS:NP 2:1 (red) and SPP NS:NP 1:2 (blue dashed) models. Bottom: SLD profile of the CER NSd7 from the SPP NS:NP 2:1 model from the experiment (black) compared with the CER NSd7 profile of the central bilayer of the three-bilayer stack from one simulation each of the SPP NS:NP 2:1 (red) and SPP NS:NP 1:2 (blue dashed) models.

Figure 5 compares the experimental SLD profiles for the SPP NS:NP 2:1 model with SLD profiles from simulations of the central bilayer for both the SPP NS:NP 2:1 and SPP NS:NP 1:2 models. The simulated protiated SLD profiles of the two SPP models are nearly identical to each other, and in good agreement with the experimental profile, peak-to-peak distances in the simulated and experimental profiles are ∼5.24 nm and ∼5.40 nm, respectively. This suggests that the simulations closely reflect the experimental structure. Also, consistent with the structural properties of the simulations described above, the different CER NS:CER NP ratios had little effect on the SLD profiles. The comparison of the simulated profiles for the two SPP models is particularly interesting since this comparison could not be studied in the experiments due to the presence of phases other than the SPP in the SPP NS:NP 1:2 model.

Figure 5 also compares simulated and experimental SLD profiles, in which the end of the CER NS sphingosine chain was deuterated (CER NSd7). In this case, peaks locating the deuterated groups are positioned at ∼±0.76 nm in the simulations in excellent agreement with the experiments at ±0.72 nm. Notably, the SLD profiles from the experiment for the SPP NS:NP 2:1 model and simulations at both CER NS:CER NP molar ratios are symmetric. This is expected for the simulations, given that ∼65% of the CERs in the four inner leaflets are in the hairpin conformation. However, in the experiments, CERs are predominantly in a linear conformation that forces an asymmetric arrangement. Thus, the symmetry observed in the experimental SLD profiles suggests that the required asymmetric structures may occur locally in mirrored configurations (in stacks) to produce an overall symmetry.

### Increased Hydrogen Bonding in the SPP NS:NP 1:2 Model

**Figure 6:**
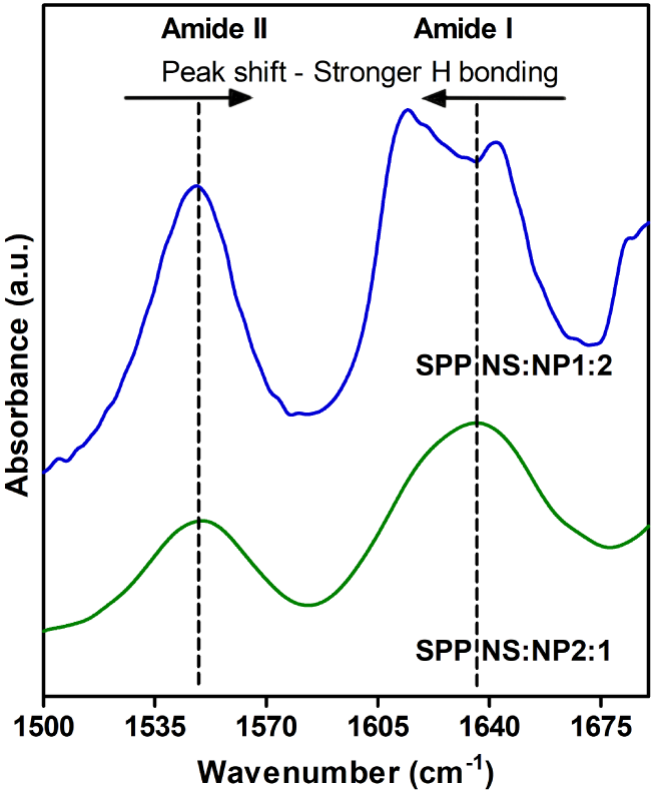
FTIR spectrum of the region 1500–1680 cm^–1^ at 10 °C, showing the amide I and II frequencies in the SPP NS:NP 2:1 (green, bottom) and SPP NS:NP 1:2 (blue, top) models. The shift of the two amide peaks indicates a difference in the hydrogen bond network in the system.

The presence of the hydroxyl and the amide group in the CER structure allows them to act as both a hydrogen bond donor and acceptor. The amide I (∼1650 cm^–1^) and amide II (∼1550 cm^–1^) vibrations measured with FTIR were used to examine hydrogen bonding in the CER headgroup regions. The amide I band results mainly from the C=O stretching vibration, and the amide II reflects primarily the N–H bending vibration and C–N stretching vibration. Stronger hydrogen bonding can be concluded when there is a lower frequency of the amide I and a higher frequency of the amide II vibrations (i.e., the positions of the two amide vibrations are closer).^100,101^ The amide I vibrations are split into two components in the SPP NS:NP 1:2 model, with a peak positioned at 1612.8 ± 0.8 cm^–1^ and another peak at 1640.7 ± 0.5 cm^–1^ (Figure 6). In the spectrum of the SPP NS:NP 2:1 model, the amide I frequency is characterized by a broad peak, centered at 1634.7 ± 2.5 cm^–1^. The peak corresponding to the amide II band has the same position in both models (1547.9 ± 0.3 cm^–1^ for the SPP NS:NP 1:2 model and 1548.1 ± 0.6 cm^–1^ for the SPP NS:NP 2:1 model).

Comparing the two systems from Figure 6, the SPP NS:NP 1:2 system displayed a lower wavenumber of the amide I vibration and a shorter distance to the amide II peak, indicating stronger hydrogen bonding compared to the SPP NS:NP 2:1 model. The intermolecular hydrogen bonding between two CER NP headgroups might contribute to the shift in position and the doublet of the amide I frequency, as it was reported to occur for pure CER NP.^102^ This difference in the hydrogen bonding of the two models is in agreement with other studies, as a stronger hydrogen bonding for CER NP compared to CER NS was previously reported for single-component systems, as well as in SPP and LPP models.^101−103^ In previous studies, it was shown that the hydrogen bonding network affects the permeability of the lipid model.^103^ It would be interesting to study whether the stronger hydrogen bonding network of the SPP NS:NP 1:2 model would result in an improved barrier function, similar to that observed for lipids forming the LPP. This will be the subject of future studies.

The number of intermolecular hydrogen bond interactions at 32 °C (305 K) was calculated for the four inner leaflets of the simulated self-assembled three-bilayer structure (Figure S3.1). In doing so, hydrogen bonding with bulk water, which dominates the two outer leaflets, is avoided and hydrogen bonding between leaflets, as well as within leaflets, can be studied. Moreover, hydrogen bonding in the leaflets without bulk water contact represents more closely the conditions in the experimental models. The headgroup regions of the four inner leaflets of the self-assembled three-bilayer membranes contained about 0.4 water molecules per lipid for both the SPP NS:NP 1:2 and 2:1 models (Table S4.1). This is consistent with experimental observations of approximately one water molecule per lipid in fully hydrated lipid models.^63^

The CER, CHOL, and FFA molecules in the two SPP models have eight hydrogen bonding sites (Figure S4.1): four in CER NS, the same four plus one more in CER NP, two in FFA C24, and one in CHOL. Hydrogen bond pairs involving the carbonyl and amide sites of the CERs are associated respectively with the amide I and amide II vibration shifts that are measured with FTIR. In addition to the lipids, the water molecules within the four inner leaflets were included in the hydrogen bonding calculations. Table S4.2 tabulates the number of each of the 42 possible hydrogen bonding pairs in the SPP NS:NP 1:2 and 2:1 models: 33 between lipids, 8 between water and a lipid, and 1 between two water molecules. Table 4 compares the CER-normalized number of hydrogen bonds that are associated with the amide I and II FTIR vibration shifts in the SPP NS:NP 1:2 and SPP NS:NP 2:1 models for CER with “all lipids” (i.e., with CER, CHOL, and FFA), and also with water. Table 4 also provides results for amide I- and amide II-associated hydrogen bonds between CER and CHOL (normalized by the number of CHOL molecules), between CER and FFA (normalized by the number of FFA molecules), and between CER and water (normalized by the number of water molecules). Table 5 lists, for each of these same three lipid classes and water, the normalized number of hydrogen bonds between “other” bonding sites that are not associated with the amide I and II vibration shifts (i.e., do not involve the C=O and N–H sites of CER) and the sum of all hydrogen bonds (i.e., amide I and II plus other). Lipid compositions of the four inner leaflets differed slightly from the nominal lipid composition. The actual numbers for each lipid class and water are listed in Table S4.1; these numbers were used to obtain the normalized hydrogen bonding results presented in Tables 4 and 5.

**Table 4:** Number of Hydrogen Bonds at 32 °C (305 K) That are Associated with the Amide I and Amide II Vibration Shifts Measured by FTIR Normalized by the Number of the Hydrogen Bonding Molecule (Column 1)^a^^,^^b^

		Amide I		Amide II		Amide I + II^c^	
		SPP NS:NP		SPP NS:NP		SPP NS:NP	
Hydrogen bonding molecule	Hydrogen bonds with	1:2	2:1	1:2	2:1	1:2	2:1
**CER**	all lipids	0.779 ± 0.015*	0.722 ± 0.021	0.479 ± 0.001*	0.459 ± 0.007	1.116 ± 0.011*	1.062 ± 0.014
	water	0.331 ± 0.022	0.334 ± 0.025	0.126 ± 0.010	0.121 ± 0.013	0.457 ± 0.031	0.456 ± 0.037
	all lipids + water	1.110 ± 0.009*	1.057 ± 0.025	0.605 ± 0.011	0.580 ± 0.019	1.573 ± 0.022*	1.517 ± 0.030
**CHOL**	CER	0.131 ± 0.006	0.139 ± 0.011	0.049 ± 0.005	0.048 ± 0.003	0.180 ± 0.003	0.187 ± 0.013
**FFA**	CER	0.175 ± 0.010	0.171 ± 0.020	0.143 ± 0.002*	0.124 ± 0.009	0.317 ± 0.012	0.295 ± 0.024
**water**	CER	0.369 ± 0.005	0.357 ± 0.018	0.140 ± 0.003*	0.129 ± 0.003	0.509 ± 0.008	0.486 ± 0.020

a Results are for the four inner leaflets of the three-bilayer membrane from the reverse-mapped atomistic simulations of the CG self-assembled membrane with CER:CHOL:FFA at a 1:0.5:1 molar ratio, for CER NS:CER NP molar ratios that match the two SPP models; data are reported as the mean and standard deviation of three replicated simulations

b An asterisk (*) identifies a model (SPP NS:NP 1:2 or 2:1) value that is statistically significantly larger than the value of the undesignated SPP NS:NP model (*p* < 0.05).

c Equal to the sum of amide I and II minus the number of CER–CER N1–O4 hydrogen bonds (Section S4), which are included in both amide I and amide II.

**Table 5:** Number of Hydrogen Bonds with Lipids and with Water That are Not Associated with Amide I and II FTIR Measurements (Other) and the Number of All Hydrogen Bonds Including Those Associated with the Amide I and II FTIR at 32 °C (305 K)^a^^,^^b^

		Hydrogen bonds per hydrogen bonding molecule			
		other (not amide I or II)		all (other + amide I + II)^c^	
		SPP NS:NP		SPP NS:NP	
Hydrogen bond pair type	Hydrogen bonding molecule	1:2	2:1	1:2	2:1
lipid–lipid	CER	1.054 ± 0.008*	0.940 ± 0.032	2.171 ± 0.019*	2.002 ± 0.036
	CHOL	0.599 ± 0.028	0.591 ± 0.023	0.779 ± 0.031	0.778 ± 0.033
	FFA	0.869 ± 0.021*	0.817 ± 0.026	1.186 ± 0.032**^†^**	1.113 ± 0.045
lipid–water	CER	0.515 ± 0.005	0.501 ± 0.033	0.972 ± 0.030	0.957 ± 0.068
	CHOL	0.341 ± 0.003	0.384 ± 0.009*	0.341 ± 0.003	0.384 ± 0.009*
	FFA	0.741 ± 0.058	0.790 ± 0.056	0.741 ± 0.058	0.790 ± 0.056
	water	1.583 ± 0.056	1.538 ± 0.050	2.091 ± 0.059	2.023 ± 0.057
water–water	water	0.438 ± 0.034	0.457 ± 0.039	0.438 ± 0.034	0.457 ± 0.039
all	CER	1.569 ± 0.009*	1.442 ± 0.034	3.143 ± 0.014*	2.959 ± 0.038
	CHOL	0.940 ± 0.026	0.976 ± 0.014	1.120 ± 0.029	1.163 ± 0.026
	FFA	1.611 ± 0.037	1.607 ± 0.031	1.928 ± 0.027	1.903 ± 0.033
	water	2.021 ± 0.022	1.994 ± 0.021	2.529 ± 0.026*	2.480 ± 0.018

a The results, normalized by the number of the hydrogen bonding molecule (column 2), are for the four inner leaflets of the three-bilayer membrane from the reverse-mapped atomistic simulations of the CG self-assembled membrane with CER:CHOL:FFA at a 1:0.5:1 molar ratio, for CER NS:CER NP molar ratios that match the two SPP models; data are reported as the mean and standard deviation of three replicated simulations.

b A model (SPP NS:NP 1:2 or 2:1) value that is statistically significantly larger than the value of the undesignated SPP NS:NP model is denoted with an asterisk (*, *p* < 0.05) or dagger (†, *p* < 0.07).

c Equal to the sum of amide I and II minus the number of CER–CER N1–O4 hydrogen bonds (Section S4), which are included in both amide I and amide II.

The total number of CER-normalized hydrogen bonds for the amide II-related sites with lipids and water was essentially the same for the SPP NS:NP 1:2 and SPP NS:NP 2:1 models (0.61 ± 0.01 and 0.58 ± 0.02, respectively). This is consistent with the FTIR experiments in which the amide II frequency was the same for both the SPP NS:NP 1:2 and 2:1 models (Figure 6). In contrast, the total number of CER-normalized hydrogen bonds with the amide I-related sites (Table 4) was statistically significantly larger (*p* < 0.05) for the SPP NS:NP 1:2 model (1.11 ± 0.01 compared with 1.06 ± 0.03), which agrees with the larger FTIR frequency shifts observed for this model in Figure 6. The CER-normalized hydrogen bond numbers for the “other” CER hydrogen bonding sites (i.e., those that are not associated with the amide I or II FTIR vibrational shifts, such as between CER hydroxide groups) were also statistically significantly larger for the SPP NS:NP 1:2 model (Table 5). Altogether, the total number of all hydrogen bonds with the CERs (i.e., all CER-lipid and CER-water hydrogen bonds) was larger for the SPP NS:NP 1:2 model (3.14 ± 0.01 compared with 2.96 ± 0.04). As expected, almost all of the increased hydrogen bonding of the SPP NS:NP 1:2 model is related to the additional hydroxide group on the sphingosine chain of CER NP compared with CER NS (identified as the O88 hydrogen bonding site in Table S4.2 and Figure S4.1). The number of hydrogen bonds with the O88 bonding site was always statistically significantly larger for the SPP NS:NP 1:2 model (Table S4.2). On average, there are about 3.1 hydrogen bonds for every CER in the four inner leaflets of the three-bilayer model (Table 5); approximately 30% of these are with water and 70% with lipids. For comparison, there were ∼1.1 hydrogen bonds with each CHOL (approximately one-third with water) and almost 2 hydrogen bonds with each FFA (∼40% with water). These results, coupled with the FTIR measurements presented in Figure 6, confirm that increasing the CER NP to CER NS ratio strengthens the hydrogen bonding network.

Normalized hydrogen bonding of water with all lipids (with or without those associated with amide I and II sites) was also larger for the SPP NS:NP 1:2 model (1.58 ± 0.06 compared with 1.54 ± 0.050 for the “other” hydrogen bonding sites and 2.09 ± 0.06 compared with 2.02 ± 0.06 for all hydrogen bonding sites), although not by a statistically significant amount (Table 5). However, the total number of normalized hydrogen bonds with water was statistically significantly larger for the SPP NS:NP 1:2 model (2.53 ± 0.03 compared with 2.48 ± 0.02), even though the number of hydrogen bonds between water molecules was not different for the two CER NS:CER NP ratios (0.44 ± 0.03 and 0.46 ± 0.04). Water is mostly hydrogen-bonded with lipids. Only about 20% of the water hydrogen bonds were with other water molecules, perhaps because water levels in the inner leaflets are so small and well distributed.

As with the other simulation results, the hydrogen bonding numbers may be affected by the smaller numbers of CERs in the linear conformation compared with the experiments for both CER NS:CER NP ratios. Additionally, there is the possibility that phase-separated CER NP in the experimental SPP NS:NP 1:2 model caused a reduced amount of CER NP in the SPP compared with the nominal composition, which is the composition used in the simulations.

## Conclusions

In this study, the impact of changing the molar ratio of CER NS and CER NP, one of the lipid compositional deviations observed in inflammatory skin diseases, was examined in lipid models that formed exclusively the SPP. A combined approach of experiments and MD simulations was used. Both the experimental and MD results show that there are almost no differences in the lipid organization and structural parameters between the SPP NS:NP 2:1 and 1:2 models. This indicates that this change in the CER composition might not contribute substantially to the alterations in the lipid organization observed in diseased skin.^30,32,33^ Both experiments and simulations indicate that there is a stronger hydrogen bond network in the SPP NS:NP 1:2 system, compared to the SPP NS:NP 2:1 model caused by the higher concentration of CER NP, which has an additional hydroxyl group in its headgroup. Furthermore, this study shows for the same SPP NS:NP 2:1 model that the neutron diffraction data indicate a symmetric arrangement of the SPP unit cell, while the FTIR results suggest an asymmetric arrangement based on the lipid domain sizes. This can be explained by the presence of two mirrored orientations of the lipids in the SPP repeating unit. A difference between the experiments and MD simulations was the CER conformation: CER NS and CER NP in the experimental models adopt primarily a linear conformation with the acyl and sphingosine chains on different sides of the headgroup, whereas in the MD simulations, only ∼35% of the CERs are found in a linear conformation. Combining the experiments with simulation data provides more detailed information about the lipid organization, lipid chain interactions, and the hydrogen bonding network.

## Supporting Information Available

Structures of the CERs used in this study; additional experimental results; proposed schematic model for the arrangement of the SPP; detailed methodology for coarse-grained molecular simulations; detailed description of analysis methods of the simulations; and additional information about the hydrogen bond data in the simulations (PDF)
