# Supplementary material for: The Sphingosine and Phytosphingosine Ceramide Ratio in Lipid Models Forming the Short Periodicity Phase: An Experimental and Molecular Simulation Study
Source: Langmuir. 2024 Jun 25;40(27):13794–809. doi: 10.1021/acs.langmuir.4c00554 (PMC11238587; doi:10.1021/acs.langmuir.4c00554)
Supplement: Supplementary file 1 — la4c00554_si_001.pdf [file la4c00554_si_001.pdf]

Supporting Information

The sphingosine and phytosphingosine ceramide ratio in lipid models forming the short periodicity phase: an experimental and molecular simulation study

Andreea Nădăban[1], Chloe O. Frame[2], Dounia El Yachioui[1], Gerrit S. Gooris[1], Robert M. Dalgliesh[3], Marc Malfois[4], Christopher R. Iacovella[2], Annette L. Bunge[5], Clare M[c]Cabe[2,6], Joke A. Bouwstra[1*]

[1] Division of BioTherapeutics, Leiden Academic Centre for Drug Research, Leiden University, Leiden, The Netherlands
[2] Department of Chemical and Biomolecular Engineering, Vanderbilt University, Nashville, TN 37235-1604, United States of America
[3] ISIS Neutron and Muon Source, Science and Technology Facilities Council, Rutherford Appleton Laboratory, Didcot, United Kingdom
[4] ALBA Synchrotron, Cerdanyola del Vallès, Barcelona, Spain
[5] Department of Chemical and Biological Engineering, Colorado School of Mines, Golden, CO 80401, United States of America
[6] School of Engineering and Physical Science, Heriot-Watt University, Edinburgh, United Kingdom

*Corresponding author

S1. Experimental Supporting Information

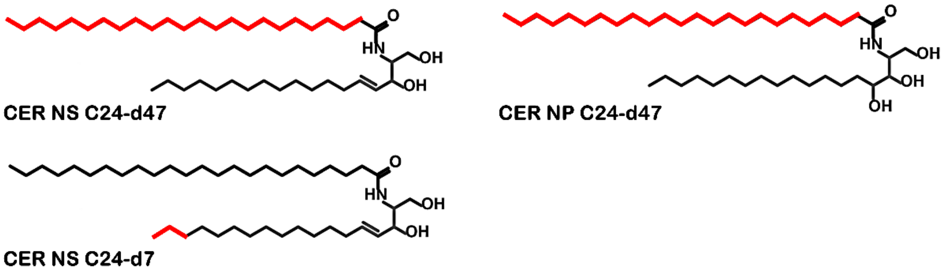

Figure S1.1. The molecular structure of the CERs used in this study. The deuterated moieties are depicted in red (the acyl chains of CER NS and CER NP and the terminally deuterated sphingosine chain of CER NS).

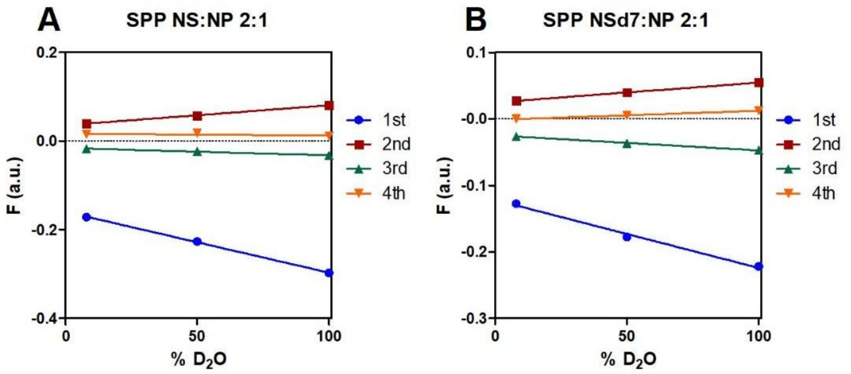

Figure S1.2. Linear fitting of the structure factors as a function of the percentage of D_2O in the D_2O/H_2O buffer for the SPP NS:NP 2:1 model and SPP NSd7:NP 2:1 models. The four diffraction orders are indicated by different symbols and colors: first (circle, blue), second (square, red), third (upward-pointing triangle, green), fourth (downward-pointing triangle, orange).

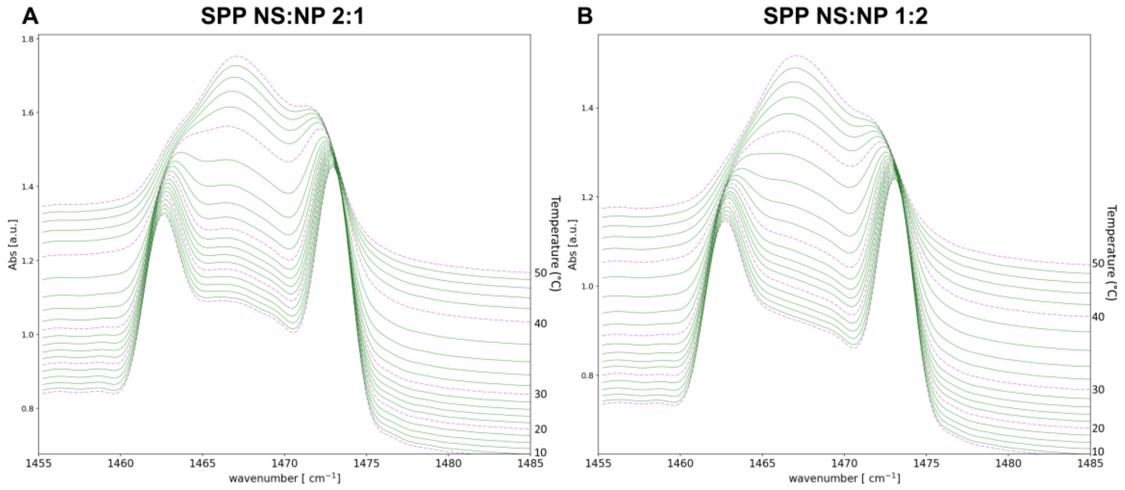

Figure S1.3. The δCH_2 vibrations of the SPP NS:NP 2:1 (A) and SPP NS:NP 1:2 (B) models, measured in the temperature range 10 – 50 °C.

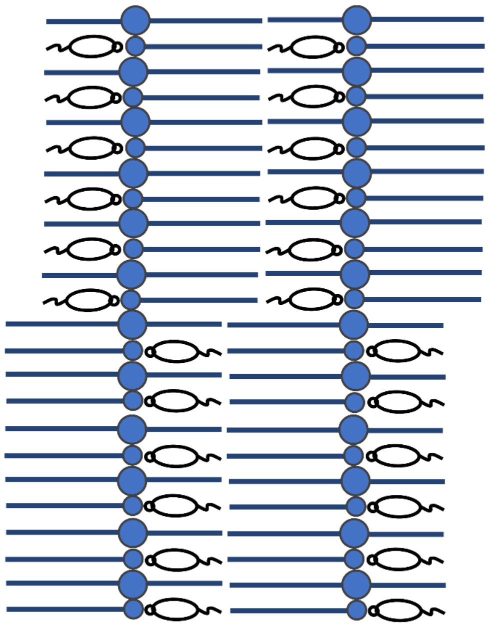

Figure S1.4. Proposed schematic model for the arrangement of the SPP unit cell. CHOL is shown in black, FFA C24 is indicated as a single acyl chain in blue and the CER is depicted in blue, in a linear conformation (the acyl chain and sphingoid base on each side of the head group). The asymmetric structure mirrors itself within the lamellar structure, resulting in a symmetric structure of the SPP unit.

S2. Coarse-grained Self-Assembly Procedure

The coarse-grained (CG) simulations were performed with the HOOMD-Blue simulation engine [1] using a 10-fs time step. The simulations were started in the constant-energy, constant-volume (NVE) ensemble at 105 K and run for 10-ps to eliminate molecular overlaps using the Velocity-Verlet integration algorithm. This was followed by another 10-ps run in the NVE ensemble at 305 K, again using the Velocity-Verlet integration algorithm. Next, the system density was equilibrated in the constant pressure and temperature (NPT) ensemble for 10 ns at 305 K and 1 bar, utilizing the Martyna, Tobias, and Klein (MTK) barostat-thermostat for integration. After this, a shape annealing process was employed to expedite self-assembly. This process, initially proposed by Moore et al. [2], entails expansion and compression of the simulation box area based on the expected area per lipid (APL_{exp}), while holding the box volume constant and operating in the constant-temperature, constant-volume (NVT) ensemble using the Nosé-Hoover thermostat to control temperature. Specifically, the diameter of the box area, a square, at the beginning of the shape annealing step was expanded by a factor of 1.58 (i.e., $2.5^{0.5}$) over 200 ns. Next, the area was decreased over 200 ns to the area at the expected diameter (L_{exp}) calculated from Eq. S2.1

$$L_{exp} = \sqrt{APL_{exp} \frac{N_{lipids}}{N_{leaflets}}} \qquad (S2.1)$$

where N_{lipids} is the number of lipids (2200) and $N_{leaflets}$ is the number of leaflets (6) in the box. The expected APL (APL_{exp}) for the CER:CHOL:FFA 1:0.5:1 molar ratio system is 0.325 nm^2. In the last step of the shape annealing process, the area diameter is held constant at L_{exp} for 200 ns. After shape annealing, the semi-isotropic barostat to NPT was re-engaged and the temperature was increased from 305 K to 450 K over 100 ns at a constant rate of heating, and then decreased back to 305 K over 50 ns at a constant rate of cooling. This temperature-annealing step serves to eliminate defects formed during the expansion/compression phase and to further relax the lipids. Finally, the simulation was run for 400 ns under physiological conditions at 305 K and 1 bar, emulating benchtop conditions. A stable equilibrated CG structure was confirmed by a constant volume simulation box over the final 200 ns. The final configuration is reverse-mapped using mBuild to regain atomic detail [3], which is then equilibrated using atomistic simulations.

S3. Simulation Data Analyses and Results

Three Bilayer (Six Leaflet) Membrane

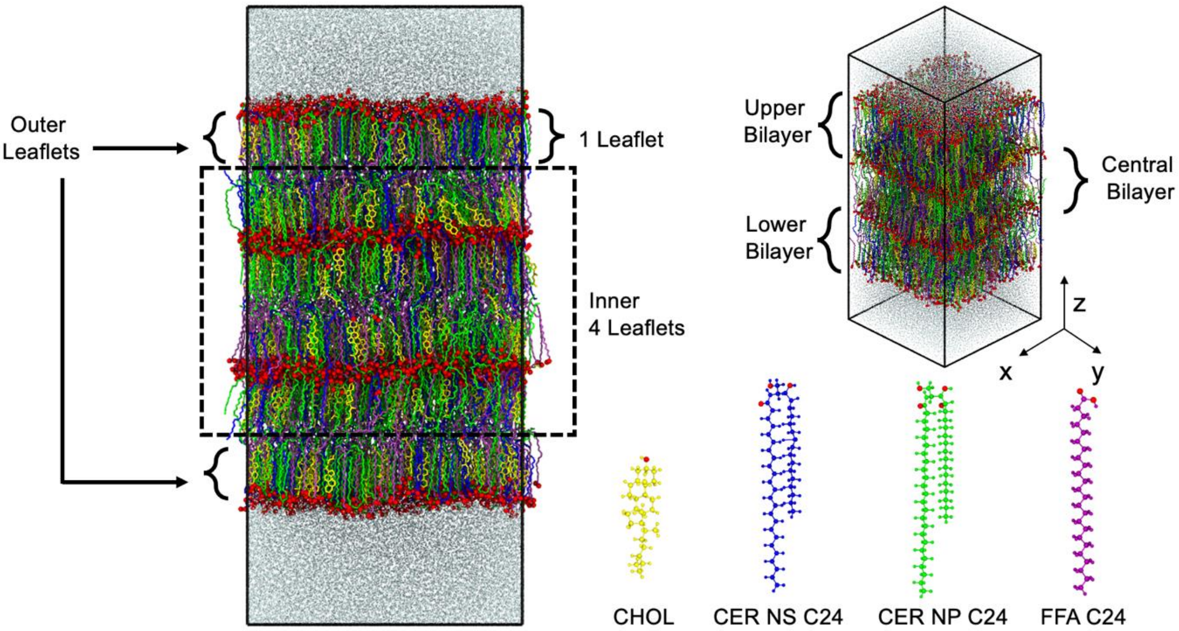

Figure S3.1. Simulation snapshot of a three-bilayer (six leaflet) membrane from the reverse-mapped atomistic simulation of the CG self-assembled CER:CHOL:FFA system at a 1:0.5:1 molar ratio representing the NS:NP 1:2 model. CER NS C24 is depicted in blue, CER NP C24 in green, CHOL in yellow, FFA C24 in purple, and water in gray on the top and bottom of the simulation box. Oxygen atoms in the lipid head groups are denoted in red, respectively. The box (black dashed line) identifies the four inner leaflets, which do not contact bulk water. The four inner leaflets were analyzed for hydrogen bonding, the number of CERs in the linear conformation, and the scattering length density profiles. The central bilayer is also shown; it was analyzed for the bilayer thickness, tilt angle, and the nematic order parameter.

Area Per Lipid

Area per lipid (APL) is calculated as

$$APL = \frac{L_x L_y}{N_{Lipids}/N_{Leaflets}}$$

where L_x and L_y are the dimensions of the simulation box in the *x* and *y* direction, respectively, and N_{Lipids} is the number of lipid head groups within the selected number of leaflets ($N_{Leaflets}$). CERs in the three-bilayer membrane are present in hairpin (with both tails in the same leaflet) and linear (with tails in adjacent leaflets) conformations. Therefore, to include both tails of every CER in the APL calculation, while excluding lipids that might be affected by contact with bulk water (i.e., those in the two outer leaflets), the APL is calculated for the four inner leaflets.

Bilayer Thickness

Bilayer thickness is calculated from the mass density profile of the three-bilayer stack, created as a histogram by binning the mass along the z-coordinate. The mass density profile for each simulation

trial is the average of the profiles from 200 frames of data collected over the last 2 ns of simulation time. The bilayer thickness is the distance between the maximum of two peaks in the average profile that are associated with the head group region of the innermost bilayer (designated as A and B in Figure S3.2) calculated using the SciPy find_peaks function [4]. Reported values of the bilayer thickness are the average of three independent simulation trials.

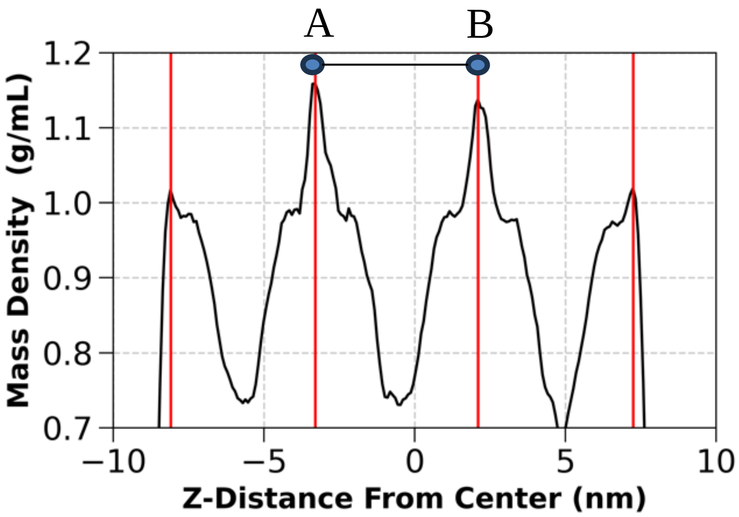

Figure S3.2. Mass density profile of all lipids in a NS:NP 2:1 model system. Points A and B identify the maximums of the inner bilayer head groups found using the SciPy find_peaks function.

Table S3.1. Structural properties calculated at 32 °C (305 K) from the reverse-mapped atomistic simulations for CG self-assembled three-bilayer membrane with CER NS:NP molar ratios matching the two SPP models in a mixture of CER, CHOL and FFA C24 with a 1:0.5:1 molar ratio. All properties were calculated for the central bilayer, except for the area per lipid, which was calculated for the four inner leaflets. Results are reported as the mean and standard deviation of three replicated simulations.

SPP Model	Bilayer Thickness (nm)	Area per lipid (nm^2)	Tilt Angle (deg)	Nematic Order Parameter (S_2)
SPP NS:NP 1:2	5.34 ± 0.05	0.334 ± 0.003	11.7 ± 0.8	0.917 ± 0.016
SPP NS:NP 2:1	5.37 ± 0.05	0.334 ± 0.001	12.0 ± 0.6	0.916 ± 0.011

Identifying CER Molecules in the Linear Conformation

For convenience, the fraction of CERs in the four inner leaflets that are in the linear conformation was determined by converting the atomistic CER structures back to CG bead mapping shown in Figure S3.3 for CER NS. A CER molecule is considered to be in the linear conformation if the angle between the TAIL-A bead on the acyl tail, the AMIDE bead in the head group, and the TAIL-S bead in the sphingosine tail is greater than 100 degrees. For each of the three simulation trials, the numbers of CER NS and NP molecules in the linear conformation, alone and together, in each of the 200 frames of data were averaged and then divided by the average number of that type of CER head groups in the inner four leaflets. The results reported in Table S3.2 are the average and standard deviation of the three simulation trials.

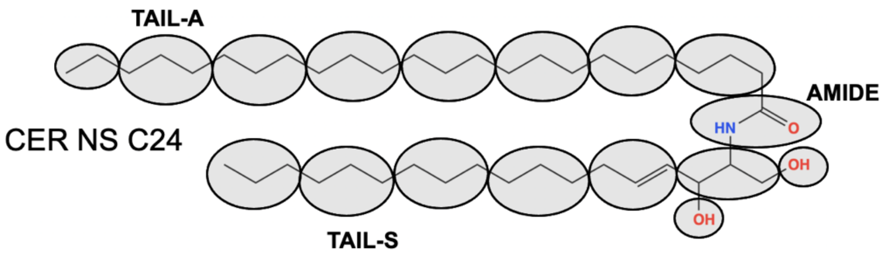

Figure S3.3. Coarse-grained mapping of CER NS C24. If the angle formed between the TAIL-A (acyl chain), AMIDE, and TAIL-S (sphingosine chain) CG beads is greater than 100 degrees, the CER is considered to be in a linear conformation.

Table S3.2. The percent of CERs NS and NP that are in the linear conformation reported as the mean and standard deviation of three replicated simulations. Results are for the four inner leaflets of the three-bilayer membrane from the reverse-mapped atomistic simulations of the CG self-assembled membrane with CER:CHOL:FFA at a 1:0.5:1 molar ratio for CER NS:NP molar ratios that match the two SPP models. [a]

**Model System**	**Linear CER NS (%)**	**Linear CER NP (%)**	**Total Linear CERs (%)**
SPP NS:NP 1:2	36 ± 3	35 ± 1	35 ± 1
SPP NS:NP 2:1	34 ± 5	39 ± 2	37 ± 3

[a] Differences between the two NS:NP ratios were not statistically significantly different for CER NS, CER NP, or CERs NS and NP combined; also, differences between CER NS and CER NP were not statistically significantly different for either NS:NP ratio ($P<0.5$).

S4. Hydrogen Bond Analysis of the Simulations

The number of intermolecular hydrogen bond interactions at 305 K were calculated for the four inner leaflets of the self-assembled three-bilayer membranes (each bilayer contains two leaflets; see Figure S3.1). CER, CHOL and FFA molecules can form hydrogen bonds associated with the eight atoms that are identified in Figure S4.1. Five of these are in the CER head groups (N1, O4, O80, O84, and O88 (in only CER NP)); two are in the FFA head group (O25 and O27), and one is in the CHOL head group (O3). Hydrogen bonds with the carbonyl (C=O) and with the N-H (nitrogen atom) in the CER molecules were assumed to be related to the observed vibration shifts in the amide I and amide II FTIR spectrum, respectively. Results reported for amide I hydrogen bonding are the sum of all hydrogen bonds with the CER C=O (i.e., O4). Amide II related hydrogen bonding results are the sum of all hydrogen bonds with CER N-H (i.e., N1). Hydrogen bonds between CER C=O and CER N-H (i.e., CER-CER N1-O4 hydrogen bonds) are included in the hydrogen bond totals for both amide I and II.

The ~ 0.4 water molecules/lipid in the head group regions of the four inner leaflets (Table S4.1) were included in the hydrogen bonding calculations. Table S4.2 lists the number for each of the 42 possible hydrogen bonding pairs in the NS:NP 1:2 and 2:1 models: 33 are between lipids, eight are between water and a lipid, and one is between two water molecules. Tables 4 and 5 in the paper report the number of hydrogen bonds for each lipid class (i.e., for CER, CHOL, and FFA) and for water normalized by the number of molecules in that lipid class and water, respectively. Thus, for CER as the designated hydrogen bonding molecule, the number of CER hydrogen bonds with lipids (i.e., with itself, CHOL, and FFA) and with water are normalized by the total number of CER NS and NP molecules in the four inner leaflets. Lipid compositions of the four inner leaflets differed slightly from the nominal lipid composition. The actual numbers for each lipid class and water are listed in Table S4.1; these numbers were used to normalize the hydrogen bonding results presented in Tables 4 and 5.

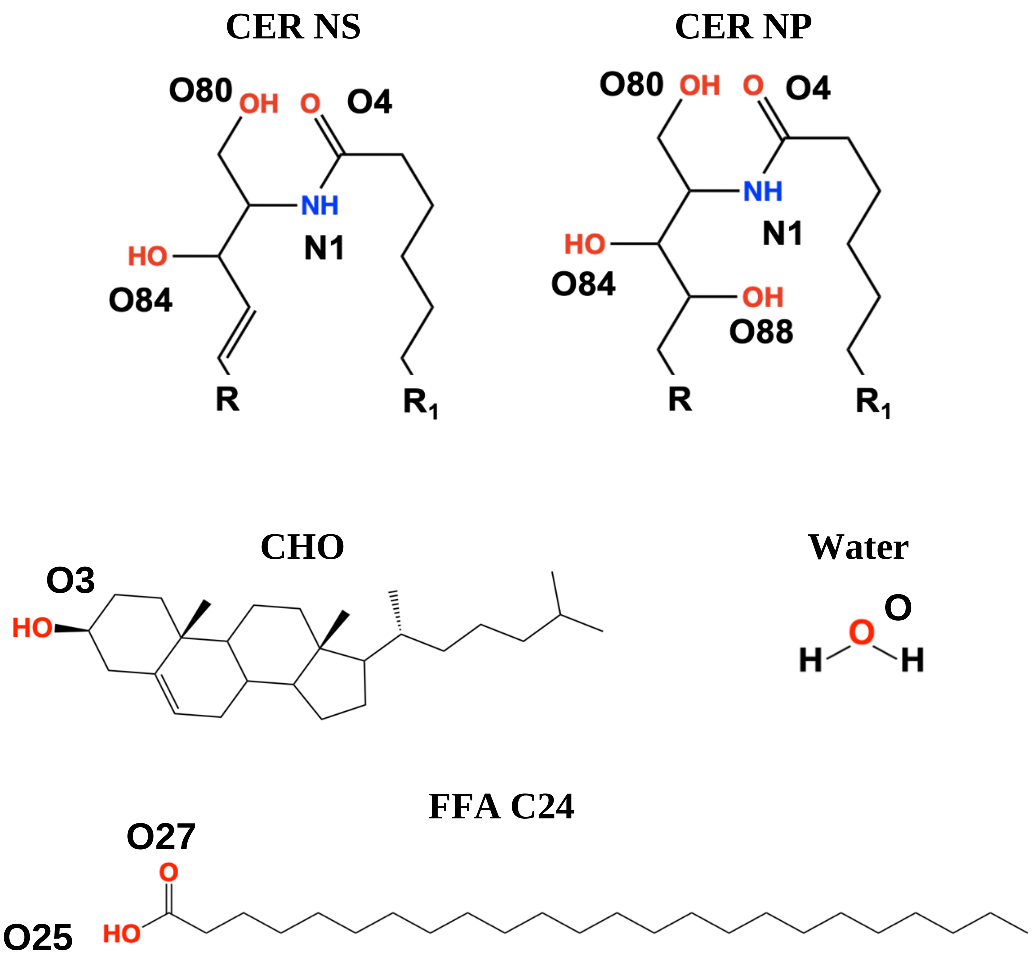

Figure S4.1. Nomenclature for the atoms in the CERs, CHOL, FFA, and water molecules that can form hydrogen bonds are specified. For the CERs, atoms participating in hydrogen bonding are N1, O4, O80, O84 and O88 (only CER NP). The O3 atom in CHOL and the O25 and O27 atoms in FFA can form hydrogen bonds. The oxygen in water is denoted as ‘O’.

Table S4.1. Number of each lipid type and water at 305 K in the four inner leaflets of the three-bilayer membranes (i.e., the leaflets that do not contact bulk water) from the reverse-mapped atomistic simulations of the CG self-assembled membranes. Results are reported as the mean and standard deviation of three replicated simulations.

**SPP NS:NP 2:1**					
Trial No.		1	2	3	All
Molecule	CER NS	390	385	396	390 ± 6
	CER NP	187	195	189	190 ± 4
	CER Total	577	580	585	581 ± 4
	CHOL	299	302	314	305 ± 8
	FFA	547	554	528	543 ± 13
	All Lipids	1423	1436	1427	1429 ± 7
	Water	543	599	495	546 ± 52
Water per Lipid		0.38	0.42	0.35	0.38 ± 0.04
Molar Ratio	NS:NP	2.09:1	1.97:1	2.10:1	2.05 ± 0.07:1
	CER:CHOL:FFA	1:0.52:0.95	1:0.52:0.96	1:0.54:0.90	1:0.53:0.94
**SPP NS:NP 1:2**					
Trial No.		1	2	3	All
Molecule	CER NS	175	196	184	185 ± 11
	CER NP	398	384	388	390 ± 7
	CER Total	573	580	572	575 ± 4
	CHOL	304	297	298	300 ± 4
	FFA	562	560	569	564 ± 5
	All Lipids	1439	1437	1439	1438 ± 1
	Water	473	533	544	517 ± 38
Water per Lipid		0.33	0.37	0.38	0.36 ± 0.03
Molar Ratio	NS:NP	1:2.27	1:1.96	1:2.11	1:2.11 ± 0.16
	CER:CHOL:FFA	1:0.53:0.98	1:0.51:0.97	1:0.52:0.99	1:0.52:0.98

Table S4.2. Number of each hydrogen bond pair calculated at 305 K for the four inner leaflets of the three-bilayer membrane (i.e., leaflets which do not contact bulk water) from the reverse-mapped atomistic simulations of the CG self-assembled membranes. Results are listed by those that are and are not associated with the amide I and II vibrations measured with FTIR (identified as Amide I, Amide II, and Other) and the sum of all hydrogen bonds reported as the mean and standard deviation of three replicated simulations. [a]

**Hydrogen Bond pair**	**Amide I**			**Amide II**			**Amide I + II** [b]		**Other**			**All** [b]	
		SPP NS:NP			SPP NS:NP		SPP NS:NP			SPP NS:NP		SPP NS:NP	
	Bond pair	1:2	2:1	Bond pair	1:2	2:1	1:2	2:1	Bond pair	1:2	2:1	1:2	2:1
**CER – CER**	O80-O4	86 ± 4	94 ± 4	N1–N1	3 ± 0	3 ± 0			O80-O80	35 ± 1	38 ± 1		
	O84-O4	99 ± 3	97 ± 5	N1–O4	82 ± 3[*]	69 ± 6			O84-O80	86 ± 1	99 ± 2[*]		
	O88-O4	43 ± 1[*]	25 ± 2	N1–O80	49 ± 3	62 ± 3[*]			O80-O88	47 ± 6[*]	25 ± 1		
	N1-O4	82 ± 3[*]	69 ± 6	N1-O84	30 ± 1	40 ± 2[*]			O84-O84	22 ± 2	32 ± 2[*]		
				N1-O88	18 ± 2[*]	10 ± 1			O84-O88	32 ± 2[*]	16 ± 2		
									O88-O88	11 ± 1[*]	4 ± 0		
	All	310 ± 3[*]	284 ± 10	All	180 ± 5	184 ± 7	409 ± 7	399 ± 11	All	233 ± 6[*]	213 ± 5	643 ± 11[*]	612 ± 7
**CER-CHOL**	O3-O4	39 ± 2	42 ± 5	N1-O3	15 ± 1	15 ± 1			O80-O3	57 ± 4	64 ± 3[†]		
									O84-O3	32 ± 2	37 ± 4		
									O88-O3	23 ± 1[*]	13 ± 2		
	All	39 ± 2	42 ± 5	All	15 ± 1	15 ± 1	54 ± 2	57 ± 6	All	111 ± 4	114 ± 5	165 ± 6	171 ± 10
**CER-FFA**	O25-O4	98 ± 6	93 ± 9	N1-O25	20 ± 1[†]	18 ± 1			O80-O27	54 ± 3	49 ± 2		
				N1-O27	60 ± 2[*]	50 ± 5			O84-O27	64 ± 4	54 ± 7		
									O88-O27	25 ± 1[*]	11 ± 2		
									O80-O25	58 ± 4	55 ± 7		
									O84-O25	36 ± 2	38 ± 4		
									O88-O25	25 ± 0[*]	12 ± 1		
	All	98 ± 6	93 ± 9	All	80 ± 1[*]	67 ± 5	179 ± 7[*]	160 ± 10	All	262 ± 6[*]	220 ± 17	440 ± 9[*]	380 ± 27
**CHOL-CHOL**									O3-O3	2 ± 0	1 ± 0		
									All	2 ± 0	1 ± 0	2 ± 0	1 ± 0
**CHOL-FFA**									O3-O25	39 ± 6	36 ± 5		
									O3-O27	27 ± 2	29 ± 1		
									All	67 ± 6	65 ± 5	67 ± 6	65 ± 5

	Amide I			Amide II			Amide I + II [b]		Other			All [b]	
**Hydrogen Bond pair**		SPP NS:NP			SPP NS:NP		SPP NS:NP			SPP NS:NP		SPP NS:NP	
	Bond pair	1:2	2:1	Bond pair	1:2	2:1	1:2	2:1	Bond pair	1:2	2:1	1:2	2:1
**FFA-FFA**									O25-O25	14 ± 2	14 ± 2		
									O25-O27	148 ± 8	144 ± 23		
									All	162 ± 9	159 ± 25	162 ± 9	159 ± 25
**All Lipid-Lipid**		448 ± 7*	420 ± 13		275 ± 3*	266 ± 2	642 ± 4*	616 ± 10		836 ± 17*	772 ± 8	1478 ± 21*	1388 ± 18
**CER-Water**	O4-O	191 ± 13	194 ± 13	N1-O	72 ± 6	70 ± 7			O80-O	115 ± 4	130 ± 13		
									O84-O	125 ± 3	131 ± 7		
									O88-O	56 ± 1*	29 ± 0		
	All	191 ± 13	194 ± 13	All	72 ± 6	70 ± 7	263 ± 19	265 ± 20	All	296 ± 5	291 ± 18	559 ± 20	556 ± 37
**CHOL-Water**									O3-O	102 ± 1	117 ± 4		
									All	102 ± 1	117 ± 4	102 ± 1	117 ± 4
**FFA-Water**									O25-O	216 ± 17	219 ± 20		
									O27-O	202 ± 18	210 ± 20		
									All	418 ± 34	429 ± 40	418 ± 34	429 ± 40
**All Lipid-Water**		191 ± 13	194 ± 13		72 ± 6	70 ± 7	263 ± 19	265 ± 20		816 ± 33	837 ± 58	1079 ± 52	1102 ± 75
**All Lipid-Lipid + Lipid-Water**		639 ± 6*	614 ± 11		348 ± 8	337 ± 9	905 ± 15†	881 ± 11		1652 ± 16	1609 ± 50	2557 ± 32	2490 ± 57
**Water-Water**									O-O	227 ± 34	251 ± 45		
									All	227 ± 34	251 ± 45	227 ± 34	251 ± 45
**Total**		639 ± 6*	614 ± 11		348 ± 8	337 ± 9	905 ± 15†	881 ± 11		1880 ± 49	1860 ± 95	2784 ± 64	2741 ± 102

[a] The value of the designated CER NS:NP model is statistically significantly larger than the value of the corresponding other CER NS:NP model ($^{*}p < 0.05$; $^{\dagger}p < 0.07$).

[b] Equal to the sum of amide I and II minus the number of CER-CER N1-O4 hydrogen bonds, which are included in both amide I and amide II.

Supporting Information References:

[1] Anderson, J.A., Glaser, J., Glotzer, S.C., HOOMD-blue: A Python package for high-performance molecular dynamics and hard particle Monte Carlo simulations, Computational Materials Science (2020), 173 109363. https://doi.org/10.1016/j.commatsci.2019.109363

[2] Moore, T.C., Iacovella, C.R., Hartkamp, R., Bunge, A.L., McCabe, C., A Coarse-Grained Model of Stratum Corneum Lipids: Free Fatty Acids and Ceramide NS, J Phys Chem B (2016), 120(37) 9944-58. https://doi.org/10.1021/acs.jpcb.6b08046

[3] Klein, C., Sallai, J., Jones, T.J., Iacovella, C.R., McCabe, C., Cummings, P.T., A Hierarchical, Component Based Approach to Screening Properties of Soft Matter, in: R.Q. Snurr, C.S. Adjiman, D.A. Kofke (Eds.), Foundations of Molecular Modeling and Simulation: Select Papers from FOMMS 2015, Springer Singapore, Singapore, 2016, pp. 79-92.

[4] Virtanen, P., Gommers, R., Oliphant, T.E., Haberland, M., Reddy, T., Cournapeau, D., Burovski, E., Peterson, P., Weckesser, W., Bright, J., van der Walt, S.J., Brett, M., Wilson, J., Millman, K.J., Mayorov, N., Nelson, A.R.J., Jones, E., Kern, R., Larson, E., Carey, C.J., Polat, I., Feng, Y., Moore, E.W., VanderPlas, J., Laxalde, D., Perktold, J., Cimrman, R., Henriksen, I., Quintero, E.A., Harris, C.R., Archibald, A.M., Ribeiro, A.H., Pedregosa, F., van Mulbregt, P., SciPy 1.0: fundamental algorithms for scientific computing in Python, Nat Methods (2020), 17(3) 261-272. https://doi.org/10.1038/s41592-019-0686-2
